# Chitosan-Based Composite Membranes with Different Biocompatible Metal Oxide Nanoparticles: Physicochemical Properties and Drug-Release Study

**DOI:** 10.3390/polym15132804

**Published:** 2023-06-24

**Authors:** Alia Baroudi, Carmen García-Payo, Mohamed Khayet

**Affiliations:** 1 Department of Structure of Matter, Thermal Physics and Electronics, Faculty of Physics, University Complutense of Madrid, Avda. Complutense s/n, 28040 Madrid, Spain; abaroudi@ucm.es; 2 Department of Industrial Engineering, Higher Polytechnic School, University Antonio Nebrija, C/Santa cruz del Marcenado 27, 28015 Madrid, Spain

**Keywords:** chitosan, metal oxide nanoparticle, swelling, mechanical properties, drug release, diffusion models, simulating gastrointestinal transit model

## Abstract

Chitosan (CS) composite membranes were prepared using different biocompatible metal oxide nanoparticles (NPs): titanium dioxide (TiO_2_); iron oxide (Fe_3_O_4_); and aluminum oxide (Al_2_O_3_). For each nanoparticle, the CS-based composite membranes were prepared with two NPs contents in the CS solution, high (H) and low (L) NPs concentrations. To establish both concentrations, the NPs saturation point in the CS polymeric matrix was determined. The influence of NP concentrations on the physicochemical properties of the CS films was assessed. The prepared CS membranes were characterized with different techniques, such as X-ray diffraction (XRD), scanning electron microscopy (SEM), and zeta potential. It was found that the addition of NPs in the CS matrix improved both swelling and mechanical properties. Nanocomposite CS membranes could be prepared using Al_2_O_3_ NPs. Swelling experiments revealed different pH-sensitive mechanisms, which might be beneficial in biomedical applications since solute permeation through CS-based composite membranes could be controlled by adjusting environmental conditions. When aspirin transport (ASA) through the prepared membranes was carried out in different release media, SGF (simulating gastric fluid) and SIF (simulating intestinal fluid without enzymes), it was observed that the Fickian diffusion coefficient (*D*) was conditioned by the pH of the release solution. In SGIT (simulating gastrointestinal transit) medium, a transition time (*t_trans_*) was detected due to the shrinkage of the CS polymeric chains, and the drug release depended not only on the Fickian’s diffusion but also on the shrinkage of the biopolymer, obeying Peppas and Sahlin equation.

## 1. Introduction

Chitosan (CS) is a linear cationic amino polysaccharide derived from chitin that can be obtained from insect exoskeletons, crustaceans shells (e.g., shrimp, prawn, crabs), as well as fungi cell walls. Structurally, CS is composed of N-acetyl-D-glucosamine and D-glucosamine units with one amino($-NH_{2}$) group and two hydroxyl (OH^−^) groups in each repeating glycosidic unit (Figure S1a in Supplementary Materials) [1]. Industrially, it is obtained from the deacetylation of chitin, the second most abundant polysaccharide in nature. Other than its biocompatibility and antibacterial properties, CS is biodegradable and non-toxic [2]. CS is insoluble at neutral and alkaline pH since its pKa is in the range of 6.2–7.0; thus, in the presence of dilute organic acids with pH values below 6, it becomes highly reactive because its −NH_2_ groups are positively charged [3]. Therefore, CS is enabled to form different types of compounds and to be one of the most used industrial polymers with applications in different areas (e.g., as an ion absorber in water treatment, food, cosmetic, medical, and pharmaceutical industries) [2,3,4,5]. One of the most current applications of CS is as a barrier for the controlled release of drugs using crosslinking agents or additives [6].

ASA (i.e., acetylsalicylic acid, C_9_H_8_O_4_, known as aspirin) is one of the drugs most consumed worldwide due to its anti-inflammatory, analgesic, antipyretic, and anticoagulant properties. Currently, one of its main uses is the prevention of cardiovascular problems, although its use is being investigated as a preventive agent for diseases, such as colon cancer and obesity [7,8]. Especially for myocardial infarction and coronary thrombosis, long-term use of low-dose ASA could significantly reduce the incidence of these diseases. However, the adverse effects of oral ingestion of ASA tablets, such as gastrointestinal disorders and bleeding, often led to treatment discontinuation [9]. The release of ASA is more effective in the intestine than in the stomach since the habitual ingestion of ASA can irritate the stomach wall causing stomach and duodenal problems [10].

The controlled release of drugs became more interesting since it improved the efficacy of drugs by reducing the number of doses, the costs of mediating, and the secondary effects on patients [11]. In this sense, the use of CS as a controlled release barrier required its crosslinking with some agents [6,12] or its combination with other polysaccharides [13] because its hydrophilic nature induced a fast swelling in aqueous media and a quick drug release [3,6,13].

The crosslinking process is commonly used to improve the chemical and mechanical stability of CS as well as to modify the controlled drug release [3]. Depending on the nature of the crosslinker, CS can be covalently or ionically crosslinked. For covalent crosslinking, agents, such as glutaraldehyde [2,3], glyoxal, or genipinin [14], can be employed. Sodium tripolyphosphate (TPP, Na_5_P_3_O_10_) is a crosslinking agent widely used for the formation of hydrogels and CS membranes by ionic interaction because of its low toxicity, high compatibility with CS [15], and ease of crosslinking [12,14,15,16]. The TPP crosslinking mechanisms can be seen in Figure S1c of Supplementary Materials.

Due to its free amino groups, CS strongly complexes with metal or metal oxide ions. It is well known that the addition of some nanoparticles (NPs) in a host polymeric matrix can modify both the physical and chemical characteristics of the matrix, improving specific properties of the resultant nanocomposite material [1,17]. In this way, the advantage of using biocompatible nanoparticles in controlled drug release has already been investigated [5,10,18,19]. Metal oxide nanoparticles (NPs) are largely claimed as efficient antibacterial agents suitable for biomedical applications. Additionally, among the many metals (oxides), ZnO, TiO_2,_ and, especially, Ag NPs were successfully added into CS polymeric matrix in several research studies [20].

In our study, different biocompatible metal oxide NPs (titanium dioxide, TiO_2_; magnetite, Fe_3_O_4_, and aluminum oxide, Al_2_O_3_) having different cationic charges have been investigated. Titanium dioxide (TiO_2_) is one of the most widely used NPs due to its high biocompatibility and anticorrosive property [5]. It is being used as a delaying agent for drug release, as a photocatalyst agent, and as a scaffold in the nanomaterials [4,11,21,22]. Magnetite (Fe_3_O_4_) is a ferromagnetic material widely considered in medical tests as a contrast agent for nuclear magnetic resonance and anticancer therapies [23]. Its use with CS is recognized in various medical applications [24], including the controlled release of drugs. Alumina or aluminum oxide (Al_2_O_3_), other than its use in controlled drug release, is employed as an adsorbent of fluorides and heavy metals [25,26].

In the present study, CS-based composite or nanocomposite membranes have been prepared by the solvent evaporation method. Previously, stable dispersions of the above-mentioned NPs in the CS solution were prepared based on the refractometry technique to determine the saturation concentration (*C_sat_*) of NPs. In this paper, two different NP concentrations were selected (one above their *C_sat_* value and another one below their *C_sat_* value). A systematic study on the effect of the type and concentration of the NP on the physicochemical properties and ASA release has been carried out. The prepared CS-based membranes were characterized in terms of X-ray diffraction, Fourier transforms infrared spectroscopy (FTIR), zeta potential, swelling, and mechanical properties. Finally, the ASA release through the prepared membranes was studied separately in both acid and basic media and in a simulating gastrointestinal transit (SGIT) media for low and high NP concentrations. Different theoretical models have been applied to study the ASA release results. In the SGIT experiment, when the pH changes from pH acid to pH basic, a transition phase is observed, showing an anomalous transport. In the present study, a complete diffusion model is proposed, and a transition time is estimated. To our knowledge, no studies have been reported yet on the development of a model for the transition from SGF to SIF media.

## 2. Materials and Methods

### 2.1. Materials

CS of high viscosity (>400 mPa.s) was purchased from Sigma-Aldrich (Saint Louis, MS, USA). Its degree of deacetylation (DDA = 86%) was determined from FTIR spectroscopy measurement [27], and its molecular weight (*M_w_ =* 1300 kDa) was obtained from viscosity measurements using the Mark–Houwink relationship [28] (i.e., the intrinsic viscosity is 0.992 mL/mg). Acetic acid, pentasodium tripolyphosphate (TPP), hydrochloric acid (HCl), ethanol, aspirin (ASA), and the necessary reagents to prepare the required buffer solutions (Na_2_HPO_4_ and NaH_2_PO_4_) were purchased from Sigma-Aldrich. The used NPs TiO_2_ (21 nm size), Fe_3_O_4_ (<50 nm size), and Al_2_O_3_ (<50 nm size) were also purchased from Sigma-Aldrich.

Two simulated fluids were prepared for the swelling test and the ASA transport experiments. The SGF solution (simulating gastric fluid), with a pH of 1.2, was a solution of 0.06M of HCl. The SIF solution (simulating intestinal fluid without enzymes), with a pH of 6.8, was prepared by mixing 46.3 mL of Na_2_HPO_4_ (1M) and 53.7 mL of NaH_2_PO_4_ (1M), adjusted up to 1 L with distilled water.

### 2.2. Membrane Preparation

The membranes were prepared by solution casting, followed by the solvent evaporation method. CS (1% *w*/*w*) was dissolved in 2% *w*/*w* acetic acid prepared with distilled water. CS solutions were mixed under stirring for 6 h using a rotor (Heidolph Model 2021, Schwabach, Germany) at a rate of 500 rpm. After CS dissolution, the obtained solution was filtered to remove any possible impurities and subsequently degassed. NPs (TiO_2_, Fe_3_O_4_, Al_2_O_3_) were then added to each CS solution. The CS-NP solutions (i.e., a solution containing each nanoparticle type) and the CS solution prepared without NPs were cast as a film on a clean glass Petri dish and then left for solvent evaporation at room temperature until they were dry. This last step varied between 3 and 8 days, depending on the NP amount in the CS solution, since the higher the concentration, the longer the drying time. For ionic crosslinking, the prepared membranes were immersed in 200 mL of a 3% *w*/*w* of TPP solution at pH 4 for 20 h, followed by thorough washing with distilled water and air drying. This crosslinking post-treatment was applied based on the results obtained in a preliminary study (see Figures S2 and S3, and Table S1 in Supplementary Materials), in which it was observed that the CS membrane (named in Supplementary Materials as CS_20h_3TPP4) exhibited stable swelling values in both simulating fluids (SGF and SIF) and improved mechanical properties. After the post-treatment, the crosslinked CS membranes were washed with distilled water and stored at room temperature until their characterization. All membranes were prepared with almost the same thickness (35 ± 5 µm). To control the membrane thickness, a previous study was carried out to relate the resulting membrane thickness with the mass of the CS solution. It was detected that the addition of the three NPs in the CS solution reduced its required amount to obtain the said thickness, between (14 ± 3)% for low NP concentration and (28 ± 5)% for high NP concentration in all the cases.

To study the effect of the NP concentration on the properties and drug release of CS-based composite membranes, two different ranges of NP concentrations (low and high concentrations) were determined. The considered NP concentrations in the CS solution were 0.5%, 2%, 5%, 9%, 17%, 33%, 41%, and 50% *w*/*w* (relative to the concentration of CS in the solution). All CS-NP dispersions were sonicated for 5 min to improve the NPs dispersion and then stirred for 12 h with a rotor. After dispersing the NPs, the CS-NP solutions were degassed for 20 min and then left to see whether there was any sedimentation of the NPs or not. Initial sedimentation started to occur for NP concentrations higher than 33% *w*/*w*. For smaller NP concentrations, stable dispersions were observed during at least 12 h. The refractometry technique was used to estimate the NP concentration from which the CS-NP solution was saturated by measuring the refractive limit angle (or critical angle, *Θ*) created at the interface between a solution and the refractometer prism. Briefly, a sample amount of each solution was taken, and the critical angle was determined at room temperature. When the CS solution with NPs began to be saturated, the critical angle tended to a stable value, indicating that the solution did not admit more NPs. From the change in the tendency of the critical angle value, the maximum NP concentration in the CS solution was estimated as explained in Supplementary Materials (Figure S4). From linear fits of the critical angle values (as can be seen in Figure S4), the saturation concentration (*C_sat_*) value of each NP could be estimated (around 8% *w*/*w* for TiO_2_ and Fe_3_O_4_ NP in the CS solution, and around 5% *w*/*w* for Al_2_O_3_ NP in the CS solution). Above this saturation concentration (*C_sat_*), the risk of sedimentation of NPs and their agglomeration could be high. Around 24 h after the addition of the NP in the CS solution, agglomeration followed by sedimentation was observed for the CS solutions prepared with TiO_2_ and Fe_3_O_4_ NP concentrations higher than 41% *w*/*w*, whereas for Al_2_O_3_ the sedimentation was observed when the Al_2_O_3_ concentration was greater than 33% *w*/*w*. Considering the *C_sat_* value for each NP, two different NP concentrations were used in this study, one below the *C_sat_* value to ensure that no NP saturation took place (called low NP concentration, L) and another one above the *C_sat_* value but lower than the NP concentration at which sedimentation was observed (called high NP concentration, H). More details are explained in Supplementary Materials (Section 2).

**Figure 1:**
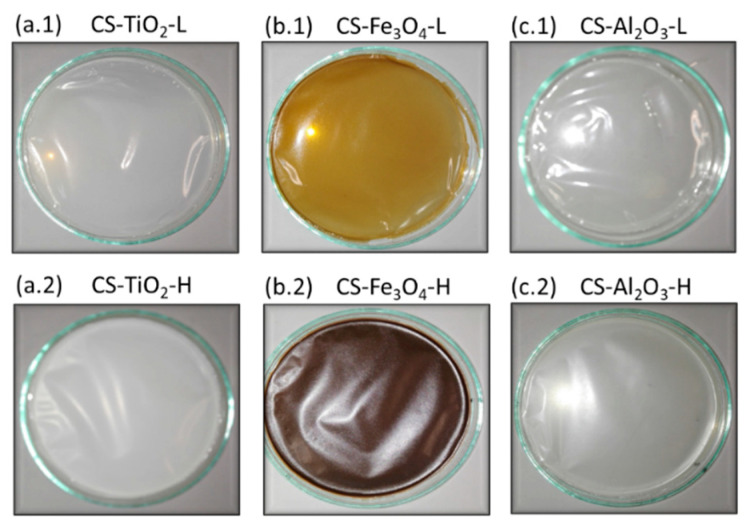
CS membranes prepared with different NPs concentrations: CS-NP-L for low NP concentration (5% *w*/*w* of TiO_2_ (**a.1**); Fe_3_O_4_ (**b.1**); and 2% *w*/*w* for Al_2_O_3_ (**c.1**)) and CS-NP-H for high NP concentration (33% *w*/*w* of TiO_2_ (**a.2**); Fe_3_O_4_ (**b.2**); and 17% *w*/*w* for Al_2_O_3_ (**c.2**)).

In the present study, CS-NP composite membranes refer to the CS-based composite membranes prepared with different NPs, while the CS membrane refers to that prepared without NPs. The CS-NP composite membranes are identified as follows: CS (denoting chitosan), followed by the NP’s name (TiO_2_, Fe_3_O_4_, and Al_2_O_3_), and finally, the type of NP concentration used with respect to the CS concentration in the CS solution (low NP concentration, L, or high NP concentration, H). For example, CS-TiO_2_-L membrane means CS composite membranes prepared with 5 % *w*/*w* TiO_2_ in the CS solution (i.e., low concentration). Photos of the prepared CS-NP membranes are shown in Figure 1.

### 2.3. Membrane Characterization

#### 2.3.1. Scanning Electron Microscopy (SEM)

The surface of the membranes was studied using the field emission scanning electron microscope (FESEM, JEOL Model JSM-6335F, Jeol Ltd., Tokyo, Japan) integrated with an Oxford Instruments EDX analyzer (model: X-Max 80 mm^2^ with a resolution of 127 eV at 5.9 KeV). Before taking the SEM images, the membrane samples were coated with a thin gold layer using a sputter-coater (Quorum model Q150RS, Judges Scientific plc, East Sussex, UK) for 90 s under 20 mA. All SEM images were taken under a voltage of 20 kV, WD 8 mm, and at 20,000 magnifications.

#### 2.3.2. X-ray Diffraction (XRD)

X-ray diffraction (XRD) spectra of the membranes were obtained using a diffractometer X’Pert-MPD (Philips, Philips, Almelo, The Netherlands) at the Cu K_α_ wavelength (*λ* = 1.54 Å). The scanning range was varied from 5° to 90° in steps of 0.04°, with a scanning speed of 1 step/s. The operating conditions were 45 kV and 40 mA using a slit of 0.15 mm.

The full width at half-maximum height (*FWHM*) of the diffraction peaks was calculated by fitting the XRD data with a Gaussian–Lorentzian function. The crystallite size, $D_{c}$, was estimated by calculating the broadening of the diffraction peaks according to the Scherrer equation,

(1) $$D_{c}=\frac{K\lambda }{FWHM\;cos\theta }$$

where *K* is the Scherrer constant or shape factor (0.9 was used in this study); 2*θ* is the diffraction angle, and *FWHM* is given in radians [29].

#### 2.3.3. Attenuated Total Reflectance/Fourier Transforms Infrared Spectroscopy (ATR-FTIR)

The FTIR spectra of the CS-based samples were obtained using a Nicolet spectrometer (model Magna-IR 750 series II), equipped with a detector DTGS-KBr (triglicerin sulfate deuterated with KBr window), a beam splitter KBr and an infrared source (Ever-Glo) employing an attenuated total reflectance (model H-ATR Multiple Bounce, Spectra Tech) with ZnSe crystal and 13 steps. The spectra were taken from 128 scans in the wavelength range 4000–400 cm^−1^ and spectral resolution of 8 cm^−1^. The absorption intensity of the peaks in this study was determined using the baseline methods.

#### 2.3.4. Mechanical Properties

The mechanical properties of the CS-based membranes were studied by a universal test device (Instron model 3366, Norwood, MA, USA) according to ASTM D 3379-75 specifications. The tensile test was performed at room temperature with a load cell of 50 N, an initial gauge length of 30 mm, and a crosshead speed of 5 mm/min. For each membrane, five samples were considered, and the tensile strength (*τ_s_*), elongation at break (*ε_b_*), and Young’s modulus (*E*) were determined as the average of five-registered data.

#### 2.3.5. Swelling

The swelling degree (*SD*) is defined as the relative quantity of liquid that a given mass of a dry sample can absorb. This was calculated by gravimetric measurements as follows [30]:

(2) $$SD\left(\%\right)=100\cdot \frac{W_{s}-W_{d}}{W_{d}}$$

where *W_s_* and *W_d_* are the weight of the swollen membrane at time *t* and the weight of the dried membrane, respectively. In this study, the membrane swelling was measured at room temperature in a simulating gastric solution (SGF) or intestinal fluids without enzymes (SIF), as indicated by the European standards for in vitro experiments [31]. The samples were removed from these solutions at regular time intervals, gently wiped with a filter paper to remove any remaining liquid drops from the surface, and then weighed on a precise balance Sartorius (Goettingen, Germany), having an accuracy of ±0.0001 g. Three samples were used for each CS-based membrane.

#### 2.3.6. Zeta Potential

The surface charge of the membranes was analyzed using streaming potential measurements with SURPASS Instrument (Anton Paar GmbH, Graz, Austria). All measurements were conducted with an adjustable-gap cell where two membrane samples of 20 mm × 10 mm were fixed on sample holders using double-sided adhesive tape. A flow channel gap of 100 µm was set between the sample surfaces. The sample holders were inserted in the adjustable-gap cell so that the membrane surfaces were facing each other. Before starting the measurement, the samples were thoroughly rinsed with the testing electrolyte (1 mM KCl aqueous solution), and the pH was adjusted to the required value using 0.1 M HCl or 0.1 M NaOH solution. All zeta potential measurements were carried out for two samples of the same membrane at 25 ± 2 °C, and the pH range was varied from 8.5 to 3.0 with a step of 0.3. Three zeta potential values were obtained for each pH value. In addition, the zeta potential of the NPs used in this study was measured at different pH values (4.0, 7.0, and 9.0) using Zetasizer Nano ZS (with disposable cells DTS1070, Malvern Instruments, Malvern, UK). Five zeta potential values were obtained for each pH value.

### 2.4. Drug Permeability Experiments

The experimental device used for drug-release tests consisted of two cylindrical cells, feed and permeate, of 100 mL separated by the membrane, as reported elsewhere [3]. The effective membrane area was (7.1 ± 0.2) cm^2^. The solution inside each cell was stirred using magnetic stirrers at 100 rpm and room temperature (22 ± 2 °C). The feed cell was filled with the drug solution 2 g/L of ASA (i.e., 2 g of ASA was first dissolved in 20 mL ethanol, and then distilled water was added up to 1 L, at pH 2.6), while the permeate cell was filled with model fluids (SGF at pH 1.2 or SIF at pH 6.8).

Two types of transport experiments were performed with ASA. Initially, the study of ASA transport through each membrane was performed, according to the pH of the permeate, by measuring the concentration of ASA released in the SGF and SIF permeate solutions during 4 h. Subsequently, simulation experiments of the human gastrointestinal (SGIT) system were performed by measuring the concentration of ASA released during 1 h in SGF medium, and then the permeate was changed to SIF, and the released ASA concentration was again measured during 4 h.

A UV spectrophotometer model Genesys 105 UV-VIS (Waltham, MA, USA) was used to analyze the change in ASA concentration with time in both the feed and permeate cells. Every 30 min, a 3 mL sample was taken from each cell for UV analysis and then returned back to its corresponding cell. The absorbance measurements were carried out at the wavelength, *λ* = 277 nm [32,33], and the ASA concentration was determined by a previously established calibration.

In order to understand the ASA transport mechanism through the CS-NP composite membranes, the experimental data were fitted to three diffusion models: Fick’s zero order; Fick’s first order; and Peppas–Sahlin [34]. The following criteria for selecting the most appropriate model depending on the release medium (SGF, SIF, or SGIT) were based on the obtained *R*^2^ values.

For drug permeability experiments, the cumulative amount of drug (ASA) transferred through the membrane during a certain time *t* per membrane area, $M\left(t\right)$, was calculated as follows:

(3) $$M\left(t\right)=\frac{C\left(t\right)V}{A}$$

where *C* is the ASA concentration (in g/L) at time *t*; *V* is the volume of the permeate cell (in L), and *A* is the effective membrane area (in m^2^). Taking into account that the initial mass of ASA in the permeate side is *M(t = 0) = 0* and, at infinite time, $M_{\infty }$ *= M(t =* ∞*)* $=M_{feed}$/2, by integrating between the time lag (*T_δ_*) and any time *t*, the relative ASA amount at time *t* can be expressed as Fick’s first-order equation [35]:

(4) $$\frac{M\left(t\right)}{M_{\infty }}=1-e^{-\frac{ADK}{VL}\left(t-T_{\delta }\right)}$$

where *D* is the diffusion coefficient of the membrane (in m^2^/s); *L* is its thickness (in m), and *K* is the partition coefficient of the drug between the membrane and the permeate, which can be included in *D* as an effective diffusion coefficient ($D_{eff})$. Equation (4) is valid in experiments when $\frac{M\left(t\right)}{M_{\infty }}\geq 0.4$ [35]. For short-time experiments, when $\frac{M\left(t\right)}{M_{\infty }}\leq 0.6$ [35], Fick’s zero order can be derived by applying a first-order Taylor series approximation of Equation (4) as follows:

(5) $$\frac{M\left(t\right)}{M_{\infty }}=\frac{AD_{eff}}{VL}\left(t-T_{\delta }\right).$$

In SGIT experiments, a pH change occurred in the permeate, going from SGF medium to SIF one, inducing, therefore, a change in the swelling behavior of the membrane. It is experimentally observed that ASA release did not follow the Fickian models (Equation (4) or Equation (5)). It is important to note that when the permeate solution was changed from SGF to SIF solution, the membrane was swelled at pH 1.2 and, consequently, a transition time (*t_trans_*) was necessary until the SGF medium liquid left the membrane, and this was swelled again by SIF solution at pH 6.8. This *t_trans_* can be estimated as follows:

(6) $$t_{trans}=\frac{V_{m}\cdot SD}{100D_{M}d_{eq}}$$

where *SD* is the swelling degree of the membrane (in %); $V_{m}$ is the membrane volume (in m^3^); $d_{eq}$ is the equivalent diameter of the membrane (in m), and *D_M_* is the mean diffusion coefficient in SGF and SIF solutions.

During this transition time (*t_trans_*), in addition to the Fickian diffusion, other phenomena also affect the drug release, such as the relaxation or contraction of the polymeric chains. Peppas and Sahlin [36] studied the power-law equation in swellable release systems and observed that the release did not depend only on the Fickian’s diffusion but also on the relaxation of the polymer. The following equation, in which the release depends on both the Fickian’s diffusion and relaxation of the polymeric membrane, was proposed [36]:

(7) $$\frac{M\left(t\right)}{M_{\infty }}=k_{1}{\left(t-t_{o}\right)}^{m}+k_{2}{\left(t-t_{0}\right)}^{2m}$$

where $t_{o}$ is the elapsed time corresponding to the change in the medium ($t_{o}$ = 60 min in this work); *m* is the exponent of the Fickian’s release; *k*_1_ is the constant related to the phenomenon of Fickian’s diffusion, and *k*_2_ is the constant related to the polymer relaxation phenomenon. This mathematical model was claimed to be valid for any geometry of the system [37]. Table 1 shows the diffusion exponent (*m*) values, which depend on the geometry of the system (whether it is a sphere, a cylinder, or a film), as well as the corresponding diffusion mechanisms that take place. In the present study, the film geometry should be considered, and consequently, *m* values from 0.5 to 1.0 could be valid.

## 3. Results

### 3.1. SEM

**Figure 2:**
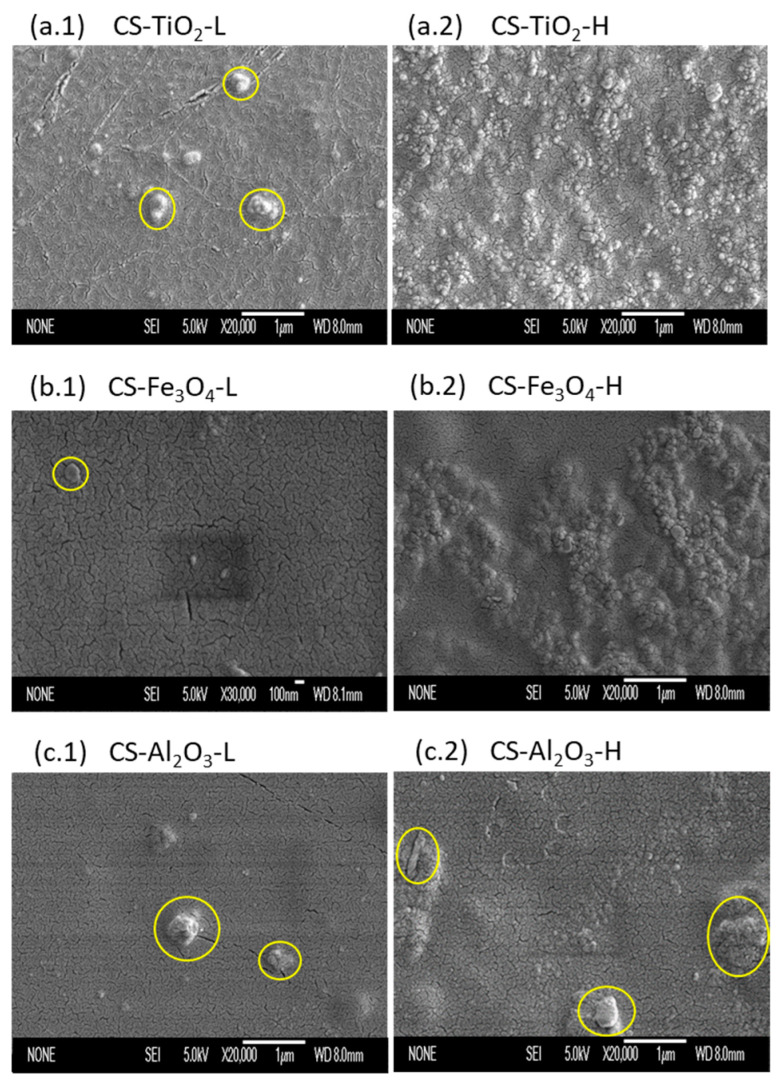
SEM images of the top surfaces of the prepared CS membranes with NPs. CS-NP-L for low NP concentrations (TiO_2_ (**a.1**); Fe_3_O_4_ (**b.1**); and Al_2_O_3_ (**c.1**)) and CS-NP-H for high NP concentrations (TiO_2_ (**a.2**); Fe_3_O_4_ (**b.2**); and Al_2_O_3_ (**c.2**)). Yellow circles indicate NPs aggregates. SEM surface images were taken at ×20,000 magnification unless the CS-Fe_3_O_4_-L image at ×30,000 magnification.

Figure 2 shows the SEM images of the top surface of the CS-NP composite membranes with both high and low NP concentrations. At low NP concentration, no significant aggregates of Fe_3_O_4_ were observed, whereas few aggregations of NPs could be detected on the CS-NP membrane surface prepared with low concentrations of TiO_2_ (Figure 2(a.1)) and Al_2_O_3_ (Figure 2(c.1)), with sizes 350 ± 5 nm and 440 ± 8 nm, respectively. These occurred, although the amounts of NPs in the dispersion were below the corresponding *C_sat_*, and both NPs together with the polymer matrix were hydrophilic, which theoretically facilitated the NPs dispersion in the solution [17]. TiO_2_ aggregates were due to the tendency of these NPs to attract each other, as observed by Díaz-Visurraga et al. [22], when preparing CS membranes with TiO_2_ nanotubes and concentrations between 0.1% *w*/*w* and 0.05% *w*/*w*.

At higher concentrations of NPs in the CS solution, above *C_sat_*, the NPs on the CS-NP membrane surface were evenly distributed despite their saturation in the CS solution. From the SEM images (see Figure 2(a.2–c.2)), it can be seen that the NP fraction becomes more extensive and bulkier, causing the smaller particles to agglomerate, reducing the overall homogeneity of the membrane. The NP dispersion in the CS solution was more uniform with the smallest particles for CS-Al_2_O_3_ membranes (except for some agglomerates, Figure 2(c.2)). The CS-TiO_2_ and CS-Fe_3_O_4_ membranes displayed small aggregates on the overall membrane surface (see, Figure 2(a.2,b.2)). Similar results were obtained by Kloster et al. [18], who prepared CS/glycerol membranes with Fe_3_O_4_ nanoparticle concentrations between 0.2–10% in the CS solution, and by Shariatinia and Nikfar [38], who prepared CS/phosphoramide membranes with Fe_3_O_4_ nanoparticle concentrations between 1 and 5% *w*/*w* of chitosan.

### 3.2. X-ray Diffraction

**Figure 3:**
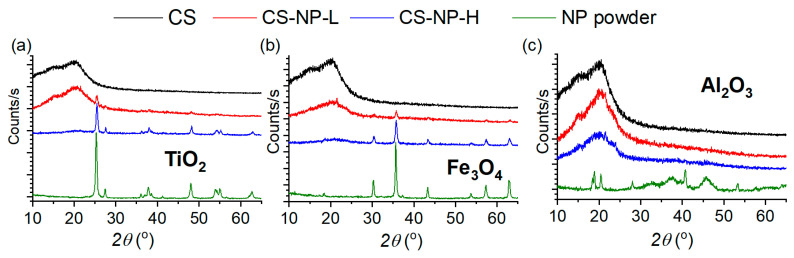
X-ray diffractograms of the CS membrane prepared without NP (black); the CS-NP membranes prepared with NPs (**a**) TiO_2_, (**b**) Fe_3_O_4,_ and (**c**) Al_2_O_3_ with low concentration (red) and high concentration (blue); and NPs (NP powder, green spectra).

Figure 3 shows the X-ray diffractograms of the NPs, the CS membrane prepared without NP, and the CS-NP membranes prepared with low and high concentrations of NPs. The characteristic peaks of the TiO_2_ NPs have been found at 2*θ* ≈ 25°, 27°, 38°, 41°, 48°, 53°, and 54°. The appearance of these peaks is due to the fact that TiO_2_ presents a mixture of anatase (at positions 25°, 38°, and 48°) and rutile (at 27° and 54°) phases [4,39,40]. The Fe_3_O_4_ NPs have the characteristic peaks at 2*θ* ≈ 31°, 35°, 43°, 54°, 57°, and 62°, which correspond to an Fe_3_O_4_ structure of spinel [41]. The Al_2_O_3_ nanoparticle spectrum has peaks at 2*θ* ≈ 18°, 19°, 20°, 27°, 32°, 37°, 40°, 45°, 53°, and 67°, which are related to the γ phase of Al_2_O_3_ [40] having a low crystallinity [42].

The CS membrane prepared without NPs exhibited two peaks, one at 2*θ* ≈ 20°, which is the characteristic peak of CS, and the other at 2*θ* ≈ 15°, which corresponds to a polymorphous crystal of hydrated CS as a complex of water and acid [3]. The presence of the NPs changed the diffraction spectrum of the CS-TiO_2_ and CS-Fe_3_O_4_ membranes (Figure 3a,b), showing the peaks of the corresponding NPs. At low concentrations, the characteristic peaks of the NPs in the CS-NP membranes were visible at 2*θ* ≈ 27° and 48° for the CS-TiO_2_ membrane (Figure 3a) and at 2*θ* ≈ 35°, 57°, and 62° for the Fe_3_O_4_ membrane (Figure 3b). At high concentrations, the characteristic peaks of the CS-TiO_2_ and CS-Fe_3_O_4_ membranes were clearly those of the corresponding NP spectrum.

From the XRD results of the prepared CS-TiO_2_ and CS-Fe_3_O_4_ membranes, it can be stated that no nanocomposites were formed [17,43], as was previously observed by Zainal et al. [4] for CS membranes prepared with TiO_2_ and by Kloster et al. [18] for CS membranes prepared with Fe_3_O_4_. Instead, CS-Al_2_O_3_ nanocomposite membranes could be formed, as can be seen from the diffractograms of these membranes that do not show any peaks relative to the Al_2_O_3_ NP [44], even at high concentrations. In addition, a left shift of the main CS peak at 2*θ* ≈ 19° (Figure 3c) was detected for the high Al_2_O_3_ concentration. This indicates that a molecular interaction between the CS polymeric chains and Al_2_O_3_ NP was taken place.

From the *FWHM* values of the main peak of the NP spectra, the average size of each NP crystallite ($D_{c}$) was calculated using Equation (1). The obtained results are summarized in Table 2, together with the size of the NPs given by the manufacturer ($D_{c,m})$. In general, the determined NP size agrees with that provided by the manufacturer for TiO_2_, Fe_3_O_4,_ and Al_2_O_3_ NPs.

### 3.3. ATR-FTIR

**Figure 4:**
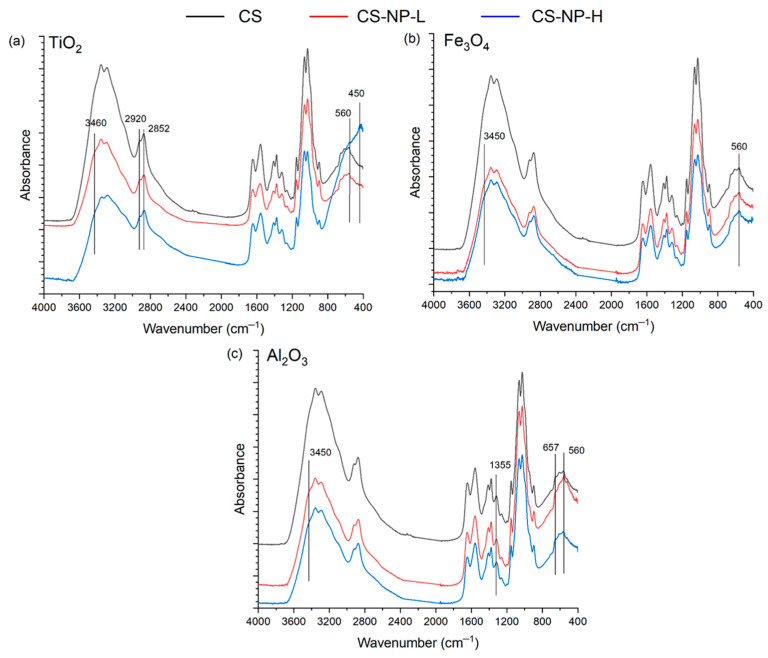
ATR-FTIR spectra of the CS membrane prepared without NP (black), the CS-NP membranes prepared with NPs (**a**) TiO_2_, (**b**) Fe_3_O_4,_ and (**c**) Al_2_O_3_ with low concentration (red) and high concentration (blue).

FTIR spectra of CS membrane and CS-NP composite membranes are shown in Figure 4. The CS membrane without NP (black lines in Figure 4a–c) exhibited a broad peak in the range of 3000–3600 cm^−1^ due to the stretching vibrations of O–H and N–H bonds. The important characteristics bands appeared at 3359 cm^−1^ (–OH stretching), 2920 and 2875 cm^−1^ (–CH stretching vibration of pyranose ring), 1650 cm^−1^ (stretching of C=O amide I band), 1590 cm^−1^ (NH_2_ in the amino group) [45], and at 1380 cm^−1^ (CH_3_ in the amide group) [46]. The peaks at 895 cm^−1^ and 1150 cm^−1^ were assigned to β(1–4) glycosidic bridge, while the bands 1065 and 1022 cm^−1^ were allocated to –C–O–C–bridge [47]. 

For CS-TiO_2_ membranes (Figure 4a), a strong band at 450 cm^−1^ was observed due to Ti–O vibration, and at 560 cm^−1^, assigned to the Ti–O–Ti bond [11,48,49], confirming the existence of TiO_2_ compound. A slight increase in bands at 2920 and 2852 cm^−1^ is a characteristic of TiO_2_–OH groups and at 3460 cm^−1^ for O–H peak from TiO_2_ [4]. For CS-Fe_3_O_4_ membranes, a peak at 560 cm^−1^ attributed to the Fe–O bond and a slight increase in the band at 3460 cm^−1^ for the O–H peak from Fe_3_O_4_ (Figure 4b) proved the existence of Fe_3_O_4_ [24,50]. The formation of the CS-Al_2_O_3_ composite was confirmed by the presence of characteristic bands at 560 and 657 cm^−1^ due to the Al–O vibration and at 1355 cm^−1^ corresponding to the Al=O bond (Figure 4c) [42,51,52,53].

### 3.4. Mechanical Properties

The results of the mechanical properties of the CS membrane and CS-NP composite membranes are summarized in Table 3. It was observed that the addition of NPs resulted in mechanical reinforcement of the CS-NP membranes. Similarly, the mechanical properties of the CS membranes were improved after their TPP post-treatment at pH4 (See Table S2 in Supplementary Materials). The TPP post-treatment reticulated the CS polymeric chains and immobilized the dispersed NPs in its matrix, obtaining CS-NP membranes with better toughness by increasing its tensile strength (*τ_s_*) and reducing its elasticity (i.e., the elongation at break (*ε_b_*) was decreased).

For all CS-NP membranes, the addition of NPs increased Young’s modulus (*E*) with respect to the CS membrane prepared without NPs. A greater improvement was detected at high NP concentrations (i.e., at low NP concentrations, the *E* values increased around 40% for the CS-TiO_2_-L membrane, 6% for the CS-Fe_3_O_4_-L membrane, and 12% for the CS-Al_2_O_3_-L membrane, whereas at high NP concentrations, the increase in *E* values were around 48%, 35%, and 29%, respectively). In general, the *τ_s_* values followed the same trend as those of *E*, being higher for the CS-NP membranes compared to those of the CS membrane prepared without NPs. Compared to the CS membrane, this increase was 53% for the CS-TiO_2_-H membrane, 17% for the CS-Fe_3_O_4_-H membrane, and 20% for the CS-Al_2_O_3_-H membrane. A greater increase in *τ_s_* values was observed when the TiO_2_ concentration was higher. This may be attributed partly to the favored interaction between TiO_2_-NP and CS polymeric chains.

According to Fu et al. [54], good adhesion between the NPs and the polymeric matrix produces an increase in *τ_s_* and *E*, as is the case of the CS-NP membranes prepared in this study, even in the presence of aggregates. Amin et al. [21] also observed an enhancement of *E* and τ_s_ values as a function of TiO_2_ concentration in CS membranes (up to 30% *w*/*w* TiO_2_ with glycerin). Guo et al. [55] explained that the addition of Al_2_O_3_ to CS hydrogels also improved the *τ_s_* of membranes prepared without these NPs.

Compared to the CS membrane prepared without NPs, the elongation at break (*ε_b_*) of all CS-NP membranes was decreased. Similarly, *ε_b_* was reduced with increasing the NP concentration. The *ε_b_* decay was greater for the CS-NP membranes prepared with high NP concentrations of TiO_2_ (67.1%) and Al_2_O_3_ (62%) (for the CS-TiO_2_-L membrane, it was 55.7%, while for the CS-Al_2_O_3_-L membrane, it was 49.4%), whereas a lower *ε_b_* reduction was observed for the CS-Fe_3_O_4_-H membrane (43%, being that of the CS-Fe_3_O_4_-L membrane only 8.9%). These results were expected since the membranes with NPs were more rigid and less elastic (i.e., more plastic to deformation) than the CS membrane prepared without NPs because the NP addition reinforces the CS polymeric chains. In fact, the presence of NPs restricted the mobility of the CS chains [18].

### 3.5. Swelling

**Figure 5:**
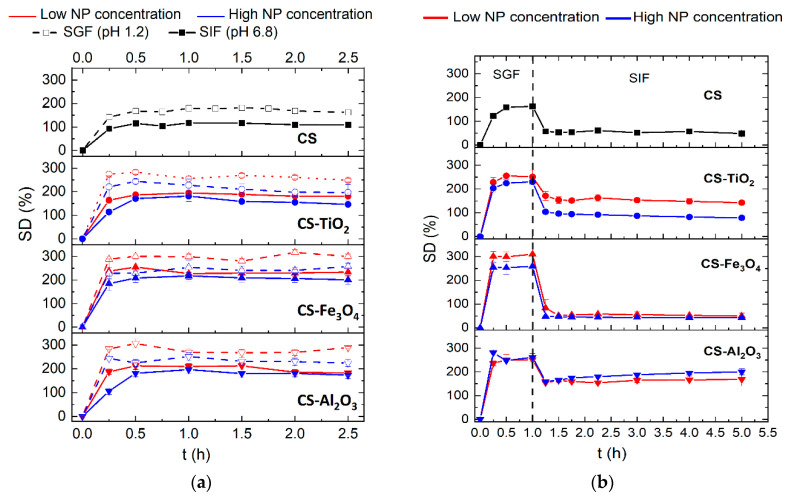
Swelling degree (*SD*) of CS membranes prepared with and without NPs as a function of time. The high NP concentrations in CS solutions are shown in blue, while the low NP concentrations are indicated in red: (●) for TiO_2_; (▲) for Fe_3_O_4_; and (▼) for Al_2_O_3_. (**a**) The dash lines and empty symbols correspond to *SD* values at pH 1.2 (SGF), and the continuous lines and filled symbols correspond to *SD* values at pH 6.8 (SIF). (**b**) *SD* values vs. time in an SGIT medium (1 h in SGF solution and then 4 h in SIF solution).

Figure 5a shows the change in the swelling degree (*SD*) with the time of the CS membrane and the CS-NP composite membranes in SGF and SIF solutions. All CS-based membranes reached their maximum swelling degree within the first 15–30 min of the experiment regardless of the pH of the solution. No significant variations in weight loss were detected in the samples during the 2.5 h of the experiment, indicating the non-degradation of the membranes in SGF and SIF solutions.

The swelling behavior in the present study is due not only to the presence of the NPs in the CS matrix but also to the post-treatment with TPP. The swelling degree for all CS-based membranes in the SGF solution was found to be higher than that in SIF solution because the CS-TPP complex is positively charged and swells in an acidic solution, whereas it shrinks in the basic solution [30]. This was also observed by Mi et al. [12] for CS beads treated with TPP due to the hydration or protonation of the free-NH_2_ groups of CS. Positively charged CS-based membranes (with and without NPs) at low pH exhibited higher *SD* values due to the fact that the repulsive force between the same positive charges of molecules caused long intermolecular distances and a more hydrophilic state. In addition, the TEM images (Figure S3(c.2) of the Supplementary Materials) showed that the interior of the CS membrane was not fully crosslinked after the TPP post-treatment. Therefore, it is expected to be the same for CS-NP composite membranes. This partly explains the observed differences in *SD* of the different membranes depending on the properties of each NP. Since the NPs used in this study also presented some solubility in the water [56,57,58,59], the *SD* values of the CS-NP composite membranes were greater than those of the CS membrane prepared without NPs (i.e., for high NP concentration in SGF solution, *SD* values increased 41% for the CS-TiO_2_-H membrane and around 60% for the CS-Fe_3_O_4_-H and CS-Al_2_O_3_-H membranes). The observed lower enhancement in the *SD* value for the CS-TiO_2_-H membrane could be due to the fact that this NP was less soluble in HCl at the experimental temperature (25 °C) without the presence of a catalyst, such as MgCl_2_ for TiO_2_ [59].

The *SD* of the CS membrane prepared without NPs was 35% lower in the SIF medium compared to that in the SGF medium (Figure 5a). The CS-NP composite membranes followed the same trend as that of the CS membrane, although the difference in *SD* values in SGF and SIF solutions was lower, around 16–30%, than that of the CS membrane being the *SD* greater for the CS-NP membranes prepared with low NPs concentrations.

As can be seen in Figure 5a, a decrease in the *SD* with the increase in NPs concentration was observed for all CS-NP composite membranes. In the SGF solution, the decrease in the *SD* value with the increase in the NPs concentration was around 17% for all CS-NP composite membranes. This trend could be due to the increase in the tensile strength (see *τ_s_* values in Table 3) as the NP concentration was increased. In fact, an increase in *τ_s_* hinders the expansion of the CS polymeric chains reducing their swelling. On the other hand, in the SIF solution, slightly different trends were observed with different NPs. The CS-TiO_2_-H and CS-Fe_3_O_4_-H membranes showed a 14–12% decrease in *SD* than those of the corresponding CS-NP membranes prepared with a low NPs concentration, CS-TiO_2_-L, and CS-Fe_3_O_4_-L membranes, respectively. However, for the CS-Al_2_O_3_-H membrane, the *SD* value barely decreased a 6% compared to the CS-Al_2_O_3_-L membrane because Al_2_O_3_ NPs formed nanocomposite with CS. A similar behavior was reported previously by Shariatinia and Nikfar [38] for CS-phosphoramide nanocomposite membranes developed with various Fe_3_O_4_ concentrations (0.005%, 2%, 23%, 28%, and 33% *w*/*w*) immersed in phosphate buffer saline (PBS) solution (0.1 M, pH 7.4). These authors observed a gradual increase in the *SD* value up to a maximum corresponding to the membrane prepared with 23% *w*/*w* of Fe_3_O_4_, followed by a decrease in the *SD* value for higher concentrations. These results indicated that the presence of NPs in the CS membrane matrix improved the relaxation of the CS polymeric chains, increasing water retention and enhancing the hydrophilicity of the CS-NP composite membranes when compared to the CS membrane prepared without NPs. Similar results were reported by Yang et al. [60] in CS membranes prepared with tetrabutyl titanate and TiO_2_ NPs (up to 10% *w*/*w* TiO_2_).

Figure 5b shows the change in the *SD* in SGIT medium (1 h in SGF solution and then 4 h in SIF solution) with time for all CS membranes prepared without and with NPs. Again, all membranes reached their maximum swelling within the first 15–30 min of the experiment in the SGF medium. For all CS membranes, a sharp decrease in the *SD* was observed when the swollen membranes were changed from SGF to SIF solutions. As was mentioned above, the *SD* is lower at pH 6.8 than at pH 1.2, and consequently, all CS membranes shrank when the medium changed. This behavior was more noticeable for the CS-Fe_3_O_4_ composite membranes. In the SGF solution, as can be seen in Figure 5b, the highest *SD* value was obtained for the CS-Fe_3_O_4_ membranes, obtaining an *SD* value of 310% for the CS-Fe_3_O_4_-L membrane, twice that of the CS membrane prepared without NPs (163%). Similar *SD* values were obtained for the CS-TiO_2_ and CS-Al_2_O_3_ membranes (around 250%). When the swollen membrane was changed to SIF solution, the highest *SD* decay was detected for the CS-Fe_3_O_4_ membranes (up to 47%).

### 3.6. Zeta Potential

The zeta potential represents the surface charge, which occurs in the presence of an aqueous solution when reactive (functional) groups dissociate on hydrophilic surfaces. Zeta potential measurements of NPs were carried out at pH 4.0, 7.0, and 9.0, and the isoelectric point (IEP) was estimated for each NP. The estimated IEP values were 6.7 ± 0.3 for TiO_2_, 6.5 ± 0.3 for Fe_3_O_4_, and 8.8 ± 0.3 for Al_2_O_3_, which are in agreement with the reported IEP values of these NPs (i.e., around 5.7–6.8 for TiO_2_ [61,62,63], 5.8–6.8 for Fe_3_O_4_ [52,61,64,65], and 8.6–9.0 for Al_2_O_3_ [61,66,67]). It is important to note that all NPs at pH 4.0 (close to the pH of the CS solution, 3.9 ± 0.1) were highly positively charged with zeta potential values of 21.5, 22.6, and 25 mV for TiO_2_, Fe_3_O_4_, and Al_2_O_3_, respectively. CS was also positively charged (since the pk_a_ of CS is 6.5), but the repelling forces, due to surface charges between NPs, are higher than NP-CS interactions, resulting in long-term stability and weaker agglomerates [68]. In addition, the highest zeta potential of Al_2_O_3_ NPs creates a powerful surface charge on the NPs, preventing the formation of aggregates. This explains the development of CS-Al_2_O_3_ nanocomposite membranes.

**Figure 6:**
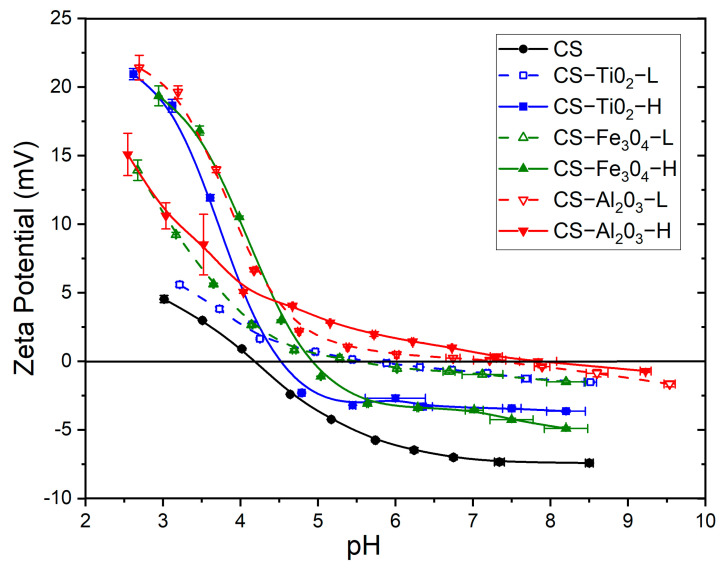
Effect of the pH on the zeta potential of the CS membranes prepared without NPs (CS, ●) and those prepared with NPs: TiO_2_ (■); Fe_3_O_4_ (▲); Al_2_O_3_ (▼) for low (open symbol and dash line) and high (solid symbol and solid line) NP concentrations. The lines are only for guidance.

Figure 6 shows the effect of the pH on the zeta potential of the CS membrane prepared without NPs and the CS-NP membranes at low and high NP concentrations. The IEP value of all CS membranes estimated from the zeta potential data are listed in Table 3. The lowest IEP value was found for the CS membrane prepared without NPs, while the highest IEP value was obtained for the CS-Al_2_O_3_ membranes. The addition of NPs in the CS membrane matrix increased its zeta potential, according to the IEP of the NPs. Similar IEP values were obtained for CS-TiO_2_ and CS-Fe_3_O_4_ membranes since the IEPs of these NPs were quite similar. Riedel et al. [69] thoroughly explained the relationship between the *SD* and zeta potential of irradiated gelatin gels and concluded that the greater the *SD*, the further the pH medium was from the IEP of the membrane due to the reduction in the ion-solvent electrostatic effect. A similar trend could be applied to our membranes in an SGF medium (pH = 1.2) when comparing the CS-NP membranes to the CS membrane. In fact, the maximum *SD* values increased from 175% for the CS membrane to the range between 220% and 300% for the CS-NP composite membranes.

When the NP concentration increased, the IEP value of the CS-TiO_2_ and CS-Fe_3_O_4_ membranes decreased because, at an acidic medium, the protonated amine groups of CS would exhibit electrostatic repulsive forces expanding the CS polymeric chains, inducing more pronounced positive charges of the NP agglomerates and, as a consequence, resulting in a lower IEP value [70]. In contrast, the increase in Al_2_O_3_ concentration enhanced the IEP value from 7.30 to 7.86. This could be due to the formation of Al_2_O_3_ CS-based nanocomposite membranes.

As can be seen in Figure 6, the zeta potential trend of the CS-TiO_2_-L and CS-Fe_3_O_4_-L membranes became flatter, with the pH enhancing their positive surface charge. Therefore, in the SIF solution (pH = 6.8), the CS-TiO_2_-L and CS-Fe_3_O_4_-L membranes are practically electroneutral. Similarly, the CS-Al_2_O_3_ membranes are almost electroneutral at pH 6.8, independent of the Al_2_O_3_ concentration in the CS solution.

### 3.7. Drug Permeability Experiments

#### 3.7.1. Transport according to pH

**Figure 7:**
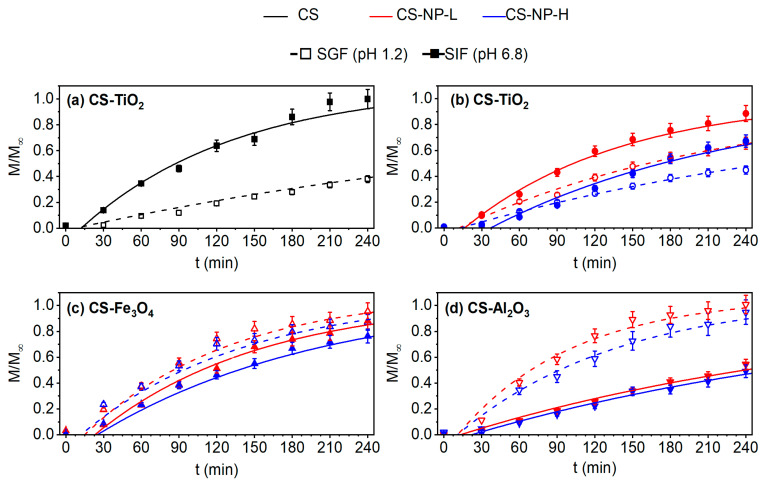
ASA transport through the CS membranes prepared without NPs ((**a**), ■) and with NPs, TiO_2_ ((**b**), ●), Fe_3_O_4_ ((**c**), ▲), and Al_2_O_3_ ((**d**), ▼) for low (red) and high (blue) NP concentrations in SGF (◯, △, ▽, and dash line) and SIF (●, ▲, ▼⤓, and solid line).

The ASA transport experiments in SGF and SIF solutions, separately, are shown in Figure 7. Different ASA release profiles were observed, depending on the pH medium and the type of NP used to prepare the CS-based composite membrane. In general, the drug release depends on the type of drug, swelling, type of NP used, and its concentration in the CS host biopolymer [11]. As can be seen in Figure 7, the diffusion of ASA through the CS-based membranes followed Fick’s model reported earlier by Equation (4). It is also observed that for a slow release, when $\frac{M\left(t=4h\right)}{M_{\infty }}\leq 0.6$, Fick’s zero order (linear fitting according to Equation (5)) can be considered. The obtained effective diffusion coefficient (*D_eff_*) and time lag (*T_δ_*) of the CS-based membranes are summarized in Table 4. It must be mentioned that the pH and ASA concentration were measured each 30 min during the transport experiment. In all experiments, the initial pH value of the feed solution was pH_ASA_ = 2.6. At the end of the experiments, their registered pH values (see Table S3 in Supplementary Materials) in both the feed and permeate solutions were changed, indicating that a reverse flow occurred through the membrane from the permeate to the feed membrane side since the transmembrane chemical potential of ASA solution tended to be the same at both sides of the membrane at a steady state. This affects the rate of ASA release through the membrane with time, as shown in Figure 7. Furthermore, no interaction between ASA and the CS-based membranes was detected (see Figure S5 in Supplementary Materials). On the other hand, no clear steady release was reached during the developed 4 h of the experiment. In the SGF solution, the achieved cumulative release varied from 38% for the CS membrane prepared without NP to 100% for the CS-Al_2_O_3_-L membrane, whereas in the SIF solution, the cumulative ASA release varied from 47% for the CS-Al_2_O_3_-H membrane to 100% for the CS membrane. Therefore, the ASA release could be controlled by adjusting the environmental conditions and NP type together with its concentration. Liu et al. [71] observed that ASA might be decomposed to salicylic acid (SA) due to the presence of both the acidic solution (pH 1.0) and the pepsin in the SGF solution. However, in our experiments, no decomposition of ASA was observed, obtaining 100% of cumulative release. In our case, the ASA solution having a pH of 2.6 (pk_a_ 3.5) was prepared with ethanol to increase its solubility [72]. Therefore, it is possible that both (pH and ethanol) can prevent the decomposition of ASA.

Both CS-TiO_2_-L and CS-TiO_2_-H membranes (Figure 7b) showed similar behavior as that of the CS membrane prepared without NP (Figure 7a) (i.e., a greater ASA diffusion in SIF than in SGF). The obtained results of the CS-TiO_2_ membranes agree with those obtained by Zang et al. [73] for the release of BSA in carboxymethylated CS/alginate blend microspheres, and by Boonsongrit et al. [74] for the release studies of CS microspheres loaded with ASA and cross-linked with TPP. When the concentration of TiO_2_ was increased, Kamari et al. [11] observed a lower ibuprofen (IBU) release in CS-TiO_2_ composite modified with methyl acrylate (MC), obtaining for a TiO_2_ concentration of 50% *w*/*w* (mass ratio of TiO_2_ to IBU/MC) a cumulative percentage of IBU release of 35% in SGF and 80% in SIF. The crystalline structure and size could be other factors to take into consideration in drug-release experiments. TiO_2_ –anatase has a smaller crystal size and degrades faster in acidic pH conditions than TiO_2_ –rutile favoring drug release as a consequence [75]. It must be noted that for the rest of the prepared CS-NP composite membranes, a greater ASA transport was observed in SGF that in the SIF medium.

The ASA release mechanism for the CS membrane prepared without NP could be explained as follows. In the SGF experiment, as the pH of the ASA solution (2.6) is lower than its pKa (3.5), the ASA remains neutral, although, in aqueous solutions, it can be solvated and could have a negative local charge density [74]. On the other hand, the CS membrane having an IEP value of 4.19 is positively charged due to the protonation of free amine groups in CS. Therefore, the ASA could bind to the swollen CS membrane surface, hindering the transport through the membrane to the permeate side (SGF solution with pH 1.2), where there is a lot of $H^{+},$ and consequently, no electrostatic force could take place. The reaction ($-{NH}_{2}$ + $H^{+}$⇆ $-{\mathrm{NH}}_{3}^{+}$) is driven at both sides of the CS membrane, raising the mutual repulsion of the charged amino group of the CS matrix [30]. Therefore, a slower ASA release was observed. In contrast, in the SIF solution (pH = 6.8), the swollen CS membrane is neutral, and the dissolved ${OH}^{-}$ groups exert an electrostatic force that enhances the ASA release significantly. Some results reported in the literature for the CS membrane prepared without NPs can be compared with the obtained ones in the present study. Kono et al. [13] prepared β-cyclodextrin-grafted carboxymethyl chitosan hydrogels using water-soluble carbodiimide as a crosslinker, obtaining, in the best case, an ASA release of 16% in SIF. Liu et al. [71], who synthesized acetylsalicylic acid-acylated chitosan (ASACTS), claimed ASA and salicylic acid (SA) release in simulated gastric fluid with an efficiency of around 60%. The release profile was fitted with the logistic and Weibull models, but no diffusion coefficient was reported, only the *R*^2^ coefficient, which was found to be 0.98, similar to our results.

Ajun et al. [76] developed CS nanoparticles crosslinked with TPP for ASA and probucol (PRO, hydrophobic drug) release and obtained an ASA release efficiency of 65% at pH = 3.0 and 87% at pH = 7.0. Luo et al. [77] prepared CS-ASA nanoparticles by interpolymer complexation and performed release studies of ASA at different pH values. The cumulative percentage of ASA release (efficiency of ASA release) reached 83% in the pH medium of 7.4, whereas it was lower, about 44%, in the pH medium of 1.2.

The IEP values of all CS-NP composite membranes were found to be much higher than the pH of ASA and pH of SGF, and their *SD* was greater than that of the CS membrane prepared without NPs. In general, the permeation increased with the *SD* enhancement. As was expected, compared with the CS membrane, an increase in *SD* involved a higher amount of ASA inside the CS-NP membrane favoring the ASA permeation until its transmembrane chemical potential became the same. The ASA release and, therefore, *D_eff_* increased as the IEP and *SD* values of the CS-NP composite membranes increased (see Table 3 and Table 4 and Figure 5a and Figure 7). These results are also consistent with the *SD* results of the CS-NP composite membranes, where the CS-TiO_2_ showed lower *SD* values in SGF than those of the CS-Fe_3_O_4_ and CS-Al_2_O_3_ membranes (see Figure 7b–d) and higher than those obtained for the CS membrane (Figure 7a). In SGF, the *D_eff_* increased in the following order compared with the CS membrane prepared without NPs: CS-TiO_2_ membranes < CS-Fe_3_O_4_ membranes < CS-Al_2_O_3_ membranes. Their *D_eff_* values were slightly lower when the NP concentration increased, as was expected since a high NP concentration in the CS matrix hinders the drug diffusion through the membrane. In addition, the IEP value of the CS-TiO_2_-H and CS-Fe_3_O_4_-H membranes was a bit lower than that of the CS-TiO_2_-L and CS-Fe_3_O_4_-L membranes, respectively. An important delay was observed before the ASA release initiation in all CS-based membranes due to their swelling delay when starting the ASA transport experiments. As can be seen in Table 4, the time lag (*T_δ_*) was delayed about 12 min for all the CS-NP composite membranes in SGF, whereas it was less delayed for the CS membrane (7 min). This may be attributed partly to the higher swelling of the CS-NP membranes and the fact that NPs hinder the ASA diffusion through the CS polymeric chains.

For the SIF experiment, at the beginning of the drug-release experiment, each side of the membrane was exposed to a medium with different pH values and, thus, exhibited quite different zeta potential values. The ASA feed solution (pH = 2.6) charged the feed side of the CS-NP membranes positively, whereas the permeate side (SIF solution at pH 6.8) was almost neutral for the CS-TiO_2_-L and CS-Fe_3_O_4_-L membranes or slightly negatively charged for the CS-TiO_2_-H and CS-Fe_3_O_4_-H membranes, as indicated by the zeta potential values plotted in Figure 6. Therefore, when compared with the CS membrane prepared without NPs, the electrostatic force between the negative local charge of ASA and the permeate surface charge of the CS-TiO_2_ and CS-Fe_3_O_4_ membranes was reduced, obtaining somewhat lower cumulative ASA release than that observed for the CS membrane (i.e., 88% for the CS-TiO_2_-L and CS-Fe_3_O_4_-L membranes, 67% for the CS-TiO_2_-H membrane, and 76% for the CS-Fe_3_O_4_-L membranes compared to 100% for CS membrane). As was expected, a slight decrease in the ASA release was detected with the increase in the NP concentration in the CS solution. For the CS-Al_2_O_3_-L and CS-Al_2_O_3_-H membranes, the zeta potential at pH 6.8 was +0.25 mV and +0.9 mV, respectively. These values indicated that the permeate side of the CS-Al_2_O_3_ membranes is neutral or slightly positively charged, reducing the electrostatic force between the negative local charge of ASA and the permeate surface charge of the CS-Al_2_O_3_ membranes and, consequently, the ASA release. In addition, the nanocomposite character of these CS-Al_2_O_3_ membranes could also indicate that stronger interaction between the CS polymeric chains and Al_2_O_3_ NPs took place, reducing the cumulative ASA release compared to that of the CS membrane (i.e., 53% for the CS-Al_2_O_3_-L membrane and 47% for the CS-Al_2_O_3_-H membrane compared to 100% for the CS membrane). Under these conditions, there was higher reverse flow between both feed and permeate solutions in SIF experiments, as can be seen in Table S3 in Supplementary Materials. The CS-Al_2_O_3_ membranes exhibited the highest feed pH at the end of the transport experiment, which agreed with its lowest ASA release. The initial difference in the chemical potential of ASA is the driving force that causes the reverse flow (${OH}^{-}$), increasing the pH of the membrane feed side.

The ASA release and, therefore, *D_eff_* decreased as the IEP approached the value of SIF pH (6.8) and as the NP concentration was increased (see Table 3 and Table 4). This behavior caused a longer time lag, *T_δ_*, in SIF compared to that obtained in SGF, especially for the CS-NP composite membranes prepared with high NP concentration (see Table 4). The time lag was more than 22 min for the CS-NP membranes prepared with high NP concentration. In the literature, few papers have been reported about polymer–metal oxide composite for ASA release. Only a comparison between the cumulative percentage release of ASA can be carried out since kinetics parameters, such as diffusion coefficients and time lag, have not been reported. Chen et al. [78] prepared polymerized glucose-coated Fe_3_O_4_ NP via the glycothermal method. A maximum aspirin release of 32% in a phosphate buffer solution (SIF) was reached within 180 min.

#### 3.7.2. Simulation Experiments of Gastrointestinal Conditions (SGIT)

**Figure 8:**
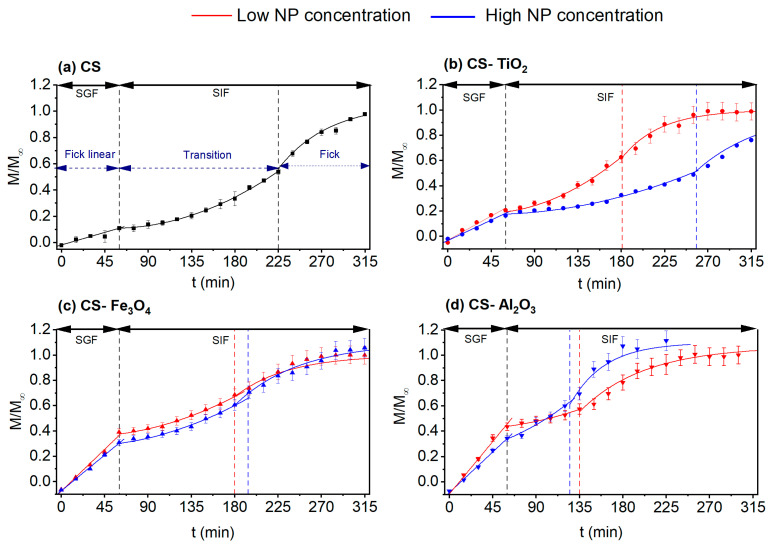
ASA transport in SGIT through the CS-based membranes prepared without NP ((**a**), ■) and with NPs, TiO_2_ ((**b**), ●) Fe_3_O_4_ ((**c**), ▲), and Al_2_O_3_ ((**d**), ▼) for low NP concentration (red) and high NP concentration (blue) in SGF medium for 60 min and then SIF medium for 4 h. The black dashed lines correspond to the change from SGF to SIF medium. The red and blue dashed lines correspond to the end of the transition phase (*t_trans_*) calculated from Equation (6) for low and high NP concentrations, respectively.

The results of ASA transport under simulated gastrointestinal conditions (SGIT) are shown in Figure 8 and summarized in Table 5 and Table 6. The ASA release in the SGF medium followed Fick’s zero-order approximation (linear fitting), as can be seen from the adjusted data in Equation (5). The obtained data during the first hour of the SGIT experiment (Table 5) were found to be comparable to those given in Table 4 (*D_eff_* values in SGF). However, when the SGF medium was changed by the SIF medium, remarkable changes in ASA release occurred. The ASA release did not follow Fick’s zero or first order but a potential relationship. This significant change in the ASA release trend was observed for all CS-based membranes, and it could be caused by the change in the pH of the permeate solution. In fact, as can be seen in Figure 5b, a considerable decrease in the *SD* of the swollen CS membranes was observed when the liquid solution was changed from acidic, SGF, solution to basic, SIF, solution. Consequently, this sharp shrinkage of the CS polymeric chains stops the ASA release, and the CS membranes need a transition time to release ASA. This transition phase, marked with dashed lines in Figure 8, can be adjusted to Peppas–Sahlin model (Equation (7)), where the ASA release depends exponentially on both Fickian’s diffusion and relaxation of the polymeric membrane. After this transition time, the ASA release returned to follow Fickian’s diffusion with a reasonable agreement to Fick’s first-order approximation (Equation (4)). However, as listed in Table 4 and Table 5, the $D_{eff}$ values obtained from the fitting to Equation (4) increased significantly compared to ASA release directly in the SIF medium. For the CS membrane prepared without NP, the $D_{eff}$ value in the only SIF solution was 3.8 × 10^−2^ m^2^/s, whereas, in the SGIT medium, the $D_{eff}$ value increased up to 8.5 × 10^−2^ m^2^/s. A considerable enhancement of $D_{eff}$ was obtained for the CS-Al_2_O_3_ nanocomposite membranes, with a 10-fold increase when the ASA release was carried out in SGIT medium for both Al_2_O_3_ concentrations (i.e., from 1.5 to 13 × 10^−2^ m^2^/s, for the CS-Al_2_O_3_-L membrane, and 1.4 to 20 × 10^−2^ m^2^/s for the CS-Al_2_O_3_-H membrane (see Table 5).

Several exponents (*m* in Equation (7)) were considered to adjust the registered data to Equation (7). The best fitting to Equation (7) (best *R*^2^ values) was obtained with *m* = 0.8. According to Table 1, the diffusion release mechanisms of ASA in the SGIT medium could be considered as an anomalous transport. The values of *k*_1_ (related to Fickian’s diffusion) and *k*_2_ (related to polymer relaxation) obtained from the experimental data-fitting Equation (7) are listed in Table 6. It is worth noting that initial adjustments were performed as proposed by other authors [79,80], but the Higuchi and Korsmeyer –Peppas equations did not fit the experimental data. The good fitting to the Peppas–Sahlin equation indicated that the ASA release mechanism through the prepared CS membranes was a kind of anomalous transport since *k*_1_ had negative values. Argin et al. [79] reported similar experiments using xanthan/CS hydrogels that released microorganisms of P. acidilactici, and the observed changes in the release were attributed to the plasticization of the polymer chains during their relaxation. Similar trends obtained in this study, Figure 8, were also found by Zhang et al. [73] for the BSA release in different pH mediums, 5.8, 6.8, and 7.4, with carboxymethylated CS/alginate blend microspheres containing BSA. Argin et al. [79] studied the release of p. acidilactici encapsulated in crosslinked xanthan/CS hydrogels in a sphere form and stated that the release due to diffusion was negligible compared to that caused by the relaxation of the CS polymeric chains. Ferrero et al. [80], by using sodium carboxymethylcellulose and hydroxypropylcellulose methylmethacrylate for theophylline drug release, proved how the polymer sphere matrix dictated the release of the drug since the observed behavior depended on whether the polymer used was sodium carboxymethylcellulose or hydroxypropylcellulose methylmethacrylate, with the Fickian diffusion, *k*_1_, being negligible when the release was in a sodium carboxymethylcellulose matrix. Islam et al. [10] synthesized a hydrogel of CS and poly(vinyl alcohol) (PVA) crosslinked with tetraethoxysilane as an enteric coating for commercial aspirin tablets. The dissolution test of enteric-coated aspirin tablet in SGF (pH 1.2) showed a 7.11% aspirin release over a period of 2 h, whereas a sustained release of the remaining aspirin (83.25%) was observed in SGF (pH 6.8). In our study, the aspirin release in SGF was higher than 11% over a period of 1 h, whereas in SIF, the aspirin release of 100% was achieved. Therefore, the Fickian contribution to the ASA release could be considered negligible compared to the relaxation of the CS polymeric chain after the sharp shrinkage when the release medium was changed from SGF to SIF during the transition time.

Finally, it should be noted that there is a very good agreement between the transition time estimated from the visual inspection of the experimental data, called *t_trans-exp_*, and the *t_trans-theoretical_*, calculated from Equation (6) where the mean effective diffusion coefficient, *D_M_*, was determined from the arithmetical mean between the $D_{eff}$ values listed in Table 5. The highest discrepancy between *t_trans-exp_* and *t_trans-theoretical_* was only 13% found for the CS-Al_2_O_3_-H membrane. This agreement between them can also be corroborated in Figure 8. The shortest transition phase corresponds to the CS-Al_2_O_3_-H membrane, which could be related to the formation of the CS-Al_2_O_3_ nanocomposite membrane. The longest transition phase detected for the CS-TiO_2_-H membrane may be attributed partly to its highest tensile strength (*τ_s_*) and its lowest elongation at break (*ε_b_*) values affecting the relaxation phenomenon as a consequence.

## 4. Conclusions

CS-NP composite membranes were prepared by adding different biocompatible metal oxide NPs, such as titanium dioxide (TiO_2_), iron oxide (Fe_3_O_4_), and aluminum oxide (Al_2_O_3_). The incorporation of NPs and their concentration in the CS matrix together with the TPP post-treatment at pH 4, which ionically crosslinked the prepared CS membranes, modified the properties of the prepared CS membranes.

The CS-NP composite membranes exhibited good dispersion at a low NP concentration, while some NP aggregates were observed at higher TiO_2_ and Fe_3_O_4_ concentrations. XRD demonstrated that the addition of Al_2_O_3_ resulted in the formation of CS-Al_2_O_3_ nanocomposites, unlike the TiO_2_ and Fe_3_O_4_ NPs. The highest zeta potential of Al_2_O_3_ NPs induced a powerful surface charge on the NP, preventing their aggregation. This could also explain the formation of CS-Al_2_O_3_ nanocomposite membranes.

The addition of NPs also affected the mechanical properties of the CS-NP membranes, improving their Young’s module and tensile strength but reducing their elasticity. The *SD* showed that the CS-NP composite membranes had a greater absorption in SGF and SIF solution than the CS membranes prepared without NPs, although for higher NP concentrations in CS solution, *SD* was reduced. In addition, the pH of the solution affected the *SD*. For all CS membranes in the SGF solution, the *SD* was found to be greater than in the SIF solution because the CS-TPP complex was positively charged and swelled in the acidic solution, whereas they shrunk in the basic solution. It was observed that the greater the *SD* value, the further the pH medium was from the IEP of the membrane since the ion-solvent electrostatic effect was reduced. Other than the type of membrane, the release of ASA depended on the pH of the release solution. A higher ASA release was detected in a basic medium than in an acidic one for both the CS membrane prepared without NPs and those CS-TiO_2_ membranes. However, the CS-Fe_3_O_4_ and CS-Al_2_O_3_ membranes exhibited an opposite behavior, obtaining a higher ASA release in a SIF medium than in an SGF medium. This could be due to a much higher *SD* value in the SGF medium for the CS-Fe_3_O_4_ membranes (around 300% and 245% for the CS-Fe_3_O_4_-L membrane and CS-Fe_3_O_4_-H membranes, respectively) than that observed for the CS-TiO_2_ membranes (around 250% and 205% for the CS-TiO_2_-L membrane and CS-TiO_2_ membranes, respectively), although their IEP values were quite similar. The CS-Fe_3_O_4_ membranes showed a lower dependence of the ASA release as a function of pH and the NPs concentration, while the CS-Al_2_O_3_ membranes were found to be almost electroneutral independently of the Al_2_O_3_ concentration at pH 6.8, and the *SD* value in SIF medium was similar to that of the CS-TiO_2_ membranes (around 180 ± 15%), obtaining the lowest ASA release in the SIF medium.

In all cases, the release of ASA followed a Fickian trend in both acidic and basic media. However, when the transport experiments were performed in SGF medium for 1 h, and then in SIF medium for 4 h (simulated gastrointestinal conditions (SGIT)), the ASA release was no longer Fickian because the pH change from pH 1.2 to pH 6.8 caused a sharp shrinkage of the CS polymeric chains, followed by a gradual relaxation and then a Fickian’s diffusion (Fick’s first-order approximation) was again observed. The transition phase showed a good agreement with the Peppas–Sahlin model, where the relaxation contribution was more important than Fickian’s diffusion, showing an anomalous transport. The different pH-sensitive mechanisms detected in CS membrane swelling, mechanical properties, surface charges, and finally, ASA release with the use of NPs resulted to be beneficial for the appropriate application of the CS-NP composite membranes in biomedical applications because solute permeation could be controlled by adjusting environmental conditions. Because of its biodegradability and biocompatibility, CS is generally considered to be safe. CS-based composites or nanocomposites commonly offer improved properties with tunable and stimulus-responsive matrices for target-specific drugs, but the current knowledge about nanomaterial safety is not sufficient. Therefore, more in vivo studies are needed to be carried out.

## Figures and Tables

**Table 1:** Values of the diffusion exponent *m* in Equation (7) for different geometries of the system and diffusion release mechanisms according to Peppas and Sahlin. Reprinted with permission from Ref. [36]. Copyright 1989, Elsevier. Reprinted with permission from Ref. [37]. Copyright 1987, Elsevier.

Diffusion Exponent (*m*)			Mechanism
Film	Cylinder	Sphere	
0.5	0.45	0.43	Fickian diffusion
0.5 < *m* < 1.00	0.45 < *m* < 0.89	0.43 < m < 0.85	Anomalous transport
1.00	0.89	0.85	Case-II transport

**Table 2:** NP size determined from the main diffraction peak of NPs (Figure 3) and Equation (1).

Nanoparticle	Position (2*θ*, °)	FWHM (2*θ*, °)	$D_{c}$ (nm)	$D_{c,m}$ (nm)
TiO_2_	25.3	0.3454	23.6 ± 0.3	21
Fe_3_O_4_	36.6	0.3070	27.2 ± 0.3	<50
Al_2_O_3_	18.8	0.2303	35.0 ± 0.3	<50

**Table 3:** Mechanical properties (Young’s modulus, *E*; tensile strength, τ_s_; elongation at break, *ε_b_*) and isoelectric point (zeta potential measurements) of the CS membranes prepared without and with NPs at low and high NP concentrations.

Membrane	*E* (GPa)	*τ_s_* (MPa)	*ε_b_* (%)	IEP (-)
CS	4.8 ± 0.6	114 ± 6	7.9 ± 0.4	4.19 ± 0.06
CS-TiO_2_-L	6.7 ± 0.4	122 ± 6	3.5 ± 0.3	5.69 ± 0.05
CS-TiO_2_-H	7.1 ± 0.5	174 ± 24	2.6 ± 0.4	4.50 ± 0.19
CS-Fe_3_O_4_-L	5.1 ± 0.6	117 ± 16	7.2 ± 0.9	5.50 ± 0.14
CS-Fe_3_O_4_-H	6.5± 0.3	133 ± 8	4.1 ± 0.5	4.90 ± 0.14
CS-Al_2_O_3_-L	5.4 ± 0.5	121 ± 10	4.0 ± 0.9	7.30 ± 0.12
CS-Al_2_O_3_-H	6.2 ± 0.2	144 ± 17	3.0 ± 0.7	7.86 ± 0.08

**Table 4:** Effective diffusion coefficient (*D_eff_*) and time lag (*T_δ_*) obtained with the fitting to Equations (4) and (6), respectively, of the ASA transport experimental data in SGF (pH = 1.2) and SIF (pH = 6.8) for the CS membranes prepared without and with NPs. *R*^2^ values are the coefficient of determination adjusted to degree of freedom.

Membrane	pH	*R* ^2^	$D_{eff}$ (10^−10^ m^2^/s)	*T_δ_* (min)
CS	1.2	0.976	1.01 ± 0.06	7 ± 1
	6.8	0.973	3.8 ± 0.3	9 ± 2
CS-TiO_2_-L	1.2	0.989	2.2 ± 0.1	12 ± 2
	6.8	0.992	3.8 ± 0.2	16 ± 4
CS-TiO_2_-H	1.2	0.991	1.3 ± 0.5	13 ± 2
	6.8	0.969	2.4 ± 0.2	37 ± 6
CS-Fe_3_O_4_-L	1.2	0.991	4.1 ± 0.2	15 ± 2
	6.8	0.989	3.6 ± 0.2	24 ± 3
CS-Fe_3_O_4_-H	1.2	0.981	3.6 ± 0.2	12 ± 2
	6.8	0.993	2.6 ± 0.1	25 ± 3
CS-Al_2_O_3_-L	1.2	0.982	5.2 ± 0.3	11 ± 2
	6.8	0.978	1.5 ± 0.1	15 ± 2
CS-Al_2_O_3_-H	1.2	0.960	3.6 ± 0.3	11 ± 2
	6.8	0.978	1.4 ± 0.1	22 ± 3

**Table 5:** Effective diffusion coefficients ($D_{eff}$) calculated from the linear and exponential Fickian models, Fick’s zero-order Equation (4) in SGF medium and Fick’s first-order Equation (5) in SIF medium for the CS-based membranes prepared with and without NPs. *R*^2^ values correspond to the coefficient of determination adjusted to degree of freedom.

Membrane	pH	Model	*R* ^2^	$D_{eff}$ (10^−10^ m^2^/s)
CS	1.2	Lineal	0.986	1.0 ± 0.1
	6.8	Exponential	0.985	8.5 ± 0.5
CS-TiO_2_-L	1.2	Lineal	0.960	2.3 ± 0.2
	6.8	Exponential	0.962	13 ± 1
CS-TiO_2_-H	1.2	Lineal	0.998	1.59 ± 0.04
	6.8	Exponential	0.985	6.8 ± 0.5
CS-Fe_3_O_4_-L	1.2	Lineal	0.977	4.0 ± 0.4
	6.8	Exponential	0.987	11.3 ± 6
CS-Fe_3_O_4_-H	1.2	Lineal	0.995	3.2 ± 0.1
	6.8	Exponential	0.959	8.1 ± 0.7
CS-Al_2_O_3_-L	1.2	Lineal	0.989	4.3 ± 0.2
	6.8	Exponential	0.964	13 ± 1
CS-Al_2_O_3_-H	1.2	Lineal	0.995	3.4 ± 0.1
	6.8	Exponential	0.965	20 ± 3

**Table 6:** Fickian release constant (*k*_1_) and polymer relaxation constant (*k*_2_), according to the Peppas–Sahlin model, Equation (7), and the transition time (*t_trans_*, Equation (6)) of the CS membranes prepared without and with NPs. The fittings to Equation (7) were carried out using the exponent *m* = 0.8.

Membrane	*R* ^2^	*k*_1_ (10^−3^ min^−0.8^)	*k*_2_ (10^−4^ min^−1.6^)	*t_trans-exp_* (min)	*t_trans-theorical_* (min)	Error (%)
CS	0.996	−1.4 ± 0.3	1.4 ± 0.1	216	207	4
CS-TiO_2_-L	0.989	−1.1 ± 0.7	2.1 ± 0.2	178	191	7
CS-TiO_2_-H	0.991	−1.8 ± 0.3	0.92 ± 0.04	270	261	3
CS-Fe_3_O_4_-L	0.998	−1.1 ± 0.2	1.53 ± 0.05	198	183	8
CS-Fe_3_O_4_-H	0.994	−1.6 ± 0.8	1.7 ± 0.1	207	214	3
CS-Al_2_O_3_-L	0.984	−1.3 ±0.7	1.9 ± 0.2	175	179	2
CS-Al_2_O_3_-H	0.977	−2 ± 1	4.2 ± 0.7	136	153	13

## Data Availability

This study did not report any data.

## Supplementary Materials

The following supporting information can be downloaded at: https://www.mdpi.com/article/10.3390/polym15132804/s1, Figure S1: Structure of (a) chitosan, CS, (b) deprotonation, and neutralization of $-{\mathrm{NH}}_{3}^{+}$ groups from CS to TPP and (c) ionic crosslinking between $-{\mathrm{NH}}_{3}^{+}$ groups of CS and TPP. Figure S2: Swelling degree (SD) of CS membranes subjected to post-treatments of TPP at pH 4 using two different TPP concentrations (3% *w*/*w* and 5% w/w, in black and red, respectively) and during two post-treatment times (3 h in solid line and 20 h in dash line): (a) *SD* in SGF medium and (b) *SD* in SIF medium. Figure S3: TEM images of the cross-section of the CS membranes: (a) without post-treatment and with a 3% *w*/*w* TPP post-treatment in pH 4 solution during (b) 3 h (CS_3h_3TPP4) and (c) 20 h (CS_20h_3TPP4). Images a.1, b.1, and c.1 correspond to the cross-section near the membrane surface, and images a.2, b.2, and c.2 correspond to the interior of the membranes. Figure S4: Critical angle (*Θc*) as a function of the NP concentration in the CS solution: TiO_2_ (●), Fe_3_O_4_ (▲), and Al_2_O_3_ (▼). Figure S5: FTIR spectra of the CS membranes prepared without NP before (black line) and after the transport experiments using different permeate media: SGF with pH_SGF_ = 1.2 (red line) and SIF with pH_SIF_ = 6.8 (blue line). For the sake of comparison, the FTIR spectrum of ASA (green line) is also shown. Table S1: Elemental composition (carbon: C, oxygen: O and phosphorus: P) of the CS membranes subjected to TPP post-treatments at pH 4 and different times, and mechanical properties (Young’s modulus, *E*; tensile strength, *τ_s_;* elongation at break, *ε_b_*). Table S2: Mechanical properties (Young’s modulus, *E*; tensile strength, *τ_s_*; elongation at break, *ε_b_*) of the CS membranes prepared with and without NPs, and with and without 3% *w*/*w* TPP post-treatment for 20 h at pH 4. Table S3: Final pH values of the feed solution after carrying out the ASA transport experiments with the simulated gastric fluid (SGF), simulated intestinal fluid (SIF), and simulated gastrointestinal transit medium (SGIT): pH_f_ is the final pH value of the feed solution that initially contained ASA, and pH_p_ is the final pH value of the permeate solution [12,16,81].
