# Supplementary material for: Chitosan-Based Composite Membranes with Different Biocompatible Metal Oxide Nanoparticles: Physicochemical Properties and Drug-Release Study
Source: Polymers (Basel). 2023 Jun 24;15(13):2804. doi: 10.3390/polym15132804 (PMC10347105; doi:10.3390/polym15132804)
Supplement: Supplementary file 1 [file polymers-15-02804-s001.zip › polymers-2440305-Supplementary.pdf]

Supplementary Materials

Chitosan-Based Composite Membranes with Different Biocompatible Metal Oxide Nanoparticles: Physicochemical Properties and Drug Release Study

Alia Baroudi[1,2], Carmen García-Payo[1]* and Mohamed Khayet[1]*

1. Selection of the adequate TPP post-treatment

The prepared membranes were subjected to a crosslinking process using sodium tripolyphosphate (TPP). Structurally, CS is composed of N-acetyl-D-glucosamine and D-glucosamine units with one amino group ($-NH_2$) and two hydroxyl groups (OH^-) in each repeating glycosidic unit, as shown in **Figure S1a**. According to pKa of CS (pKa ~ 6.3), the dissolved CS in a low acid medium is protonated, presented $-NH_3^+$ groups and has an acidic pH. The reaction between CS and TPP is known to be pH-dependent of the TPP solution [1,2]. By adjusting the pH value of TPP solution from its original value (pH 8.6) to a value lower than 4, the mechanism of liquid curing can be changed from neutralization to ionic crosslinking [1]. When the TPP is dissolved in water (pH 9), both hydroxyl groups (OH^-) and tripolyphosphoric ions ($P_3O_{10}^{5-}$ and $HP_3O_{10}^{4-}$) coexist. The $P_3O_{10}^{5-}$ and $HP_3O_{10}^{4-}$ ions compete with the OH^- to react ionically with the $-NH_3^+$ groups of the CS by deprotonation and neutralization (**Figure S1b**) or ionic crosslinking (**Figure S1c**) resulting in a weak crosslinking. However, when the pH of the TPP is adjusted to an acid pH value (pH = 4), only tripolyphosphoric ions existed and a highly ionic crosslinking between the $-NH_3^+$ groups and the TPP anions is formed. Therefore, the crosslinking density of chitosan-TPP membranes could be improved by the modification of both in-liquid curing time and TPP concentration.

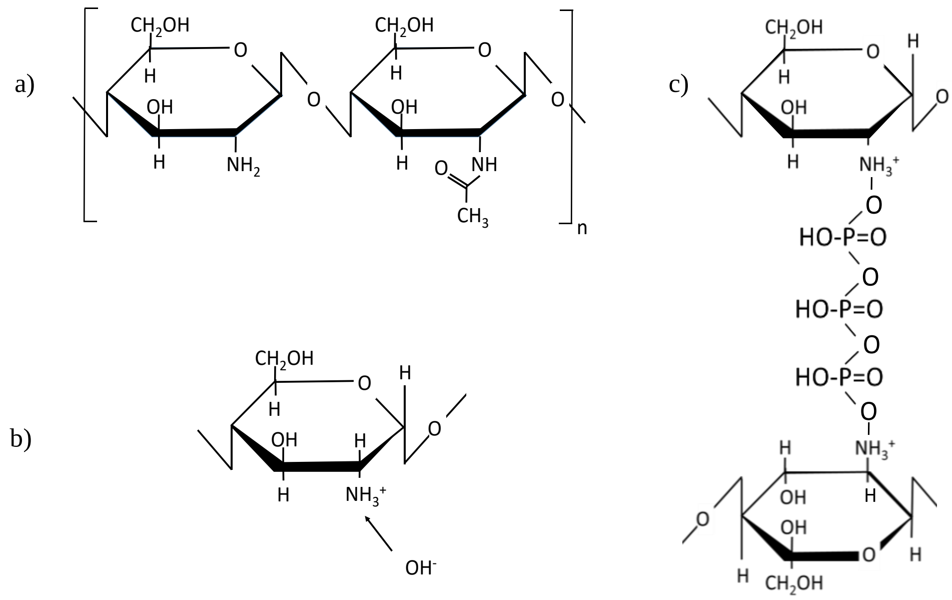

Figure S1: Structure of: (a) chitosan, CS, (b) deprotonation and neutralization of $-NH_3^+$ groups from CS to TPP and (c) ionic crosslinking between $-NH_3^+$ groups of CS and TPP.

Different post-treatments of TPP were carried out for the CS membrane prepared without NP, looking for the one that allowed the lowest release of ASA in SGF medium (pH 1.2). A convenient proof of crosslinking is the swelling behavior of the CS membranes in various pH values of aqueous media. Higher ionic crosslinking density of CS membranes resulted in less membrane swelling ability due to their high stability in acid. Previously, the post-treatment of TPP in a basic pH medium was

discarded, because in SGF the membrane was rapidly degraded and therefore it was not adequate for the swelling measurement. Moreover, when the transport experiments were carried out with that membrane, the release of ASA was 100% in less than 30 min. In contrast, when applying the TPP post-treatments at pH 4, the membranes in SGF medium did not degrade although they were kept in this medium 4 h. To estimate the best TPP crosslinking, the CS membranes were immersed in TPP solutions at two different concentrations, 3% w/w and 5% w/w of TPP at pH 4, and at different times, 3 and 20 h. It was found that for these concentrations, the two membranes showed similar swelling degree (*SD*) values in SGF medium (about 175 ± 10%) and comparable to *SD* values in SIF medium as can be seen in **Figure S2**. Therefore, the concentration 3% w/w was selected.

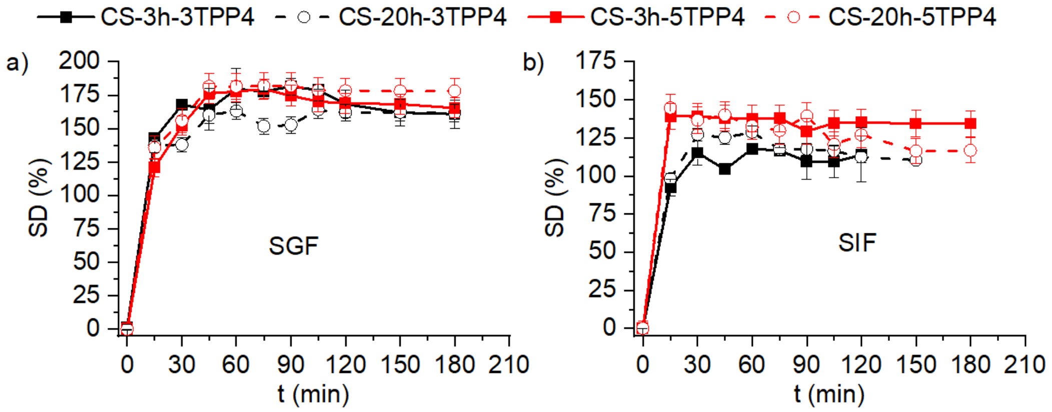

Figure S2: Swelling degree (SD) of CS membranes subjected to post-treatments of TPP at pH 4 using two different TPP concentrations (3% w/w and 5% w/w, in black and red, respectively), and during two post-treatment times (3 h in solid line and 20 h in dash line): (a) *SD* in SGF medium and (b) *SD* in SIF medium.

In addition, the elemental composition along the cross-section of the membranes were analyzed by energy dispersive spectroscopy (EDS) in order to study the TPP crosslinking of the CS membranes. It was observed that the total crosslinking was achieved after 10 min of post-treatment because phosphorous composition was almost kept constant along the cross-section of the CS membrane for 10 min, 3h and 20 h post-treatment time (see **Table S1**).

Table S1: Elemental composition (carbon: C, oxygen: O and phosphorus: P) of the CS membranes subjected to TPP post-treatments at pH 4 and different times, and mechanical properties (Young's modulus, *E*; tensile strength, τ_s; elongation at break, ε_b).

Membrane	Composition (%)						Mechanical properties		
	C	O	Na	P	Cl	Ratio P/C	*E* *(GPa)*	τ_s *(MPa)*	ε_b *(%)*
CS	62.59	31.54	0.01	0.00	0.01	0.00	2.5 ± 0.2	86 ± 10	18 ± 3
CS_1min_3TPP4	41.31	51.13	0.67	5.11	1.78	0.12	-	-	-
CS_10min_3TPP4	37.64	49.93	2.32	9.23	0.88	0.24	-	-	-
CS_3h_3TPP4	39.47	50.49	1.30	7.98	0.76	0.20	3.6 ± 0.2	90 ± 5	10 ± 2
CS_20h_3TPP4	37.71	53.17	0.56	8.40	0.16	0.22	4.8 ± 0.6	114 ± 6	7.9 ± 0.4
CS_3h_5TPP4	35.55	54.78	0.12	9.54	-	0.27	-	-	-
CS_20h_3TPP9	48.70	50.38	0.64	0.28	0.16	0.01	3.6 ± 0.5	105 ± 14	32 ± 7

The TPP post-treatment at pH 4 during more than 10 min did not modify the swelling or drug release properties of the CS membranes, but at longer post-treatment times the mechanical properties of the CS membranes were increased (see **Table S1**). Young´s modulus (*E*) was improved by 92% and the tensile strength (τ_s) was increased by 32%, whereas the elongation at break (ε_b) was reduced by 83%. Similar results were reported by Velickova et al. [3] for chitosan/TPP films.

Transmission electron microscopy (TEM, JEOL JEM 1010) was also used to study the TPP crosslinking of the CS membranes. **Figure S3** shows the TEM images of the CS membrane without post-treatment and the membrane prepared with 3% w/w TPP post-treatment for 3 and 20 h. The obtained TEM images (**Figure S3-a.1, b.1 and c.1**) near the membrane surface show a reticulation of the CS chain being higher for a longer curing time. However, the TEM images of the interior of the CS membranes (**Figure S3-a.2, b.2 and c.2**) indicate that these membranes are not entirely crosslinked. Taking into account the improvement of the mechanical properties and TEM images, the 3% w/w TPP post-treatment at pH 4 during 20 h was considered as an adequate crosslinking strategy.

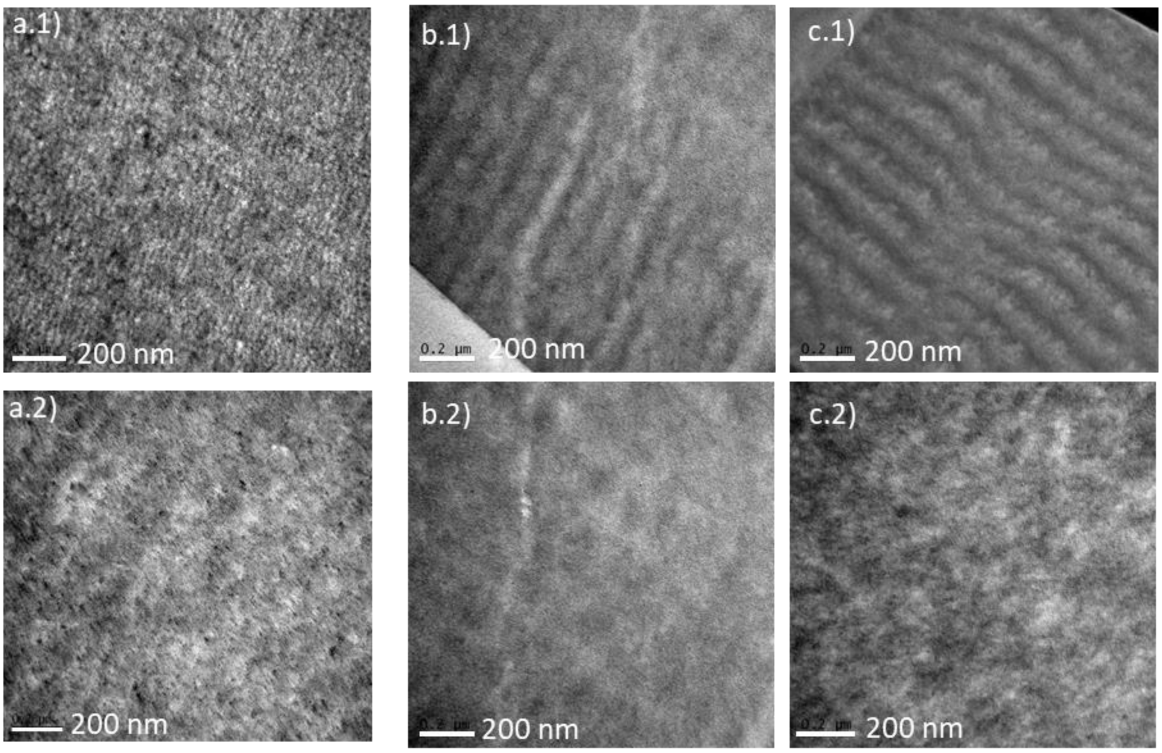

Figure S3: TEM images of the cross-section of the CS membranes: (a) without post-treatment and with a 3% w/w TPP post-treatment in pH 4 solution during (b) 3 h (CS_3h_3TPP4) and (c) 20 h (CS_20h_3TPP4). Images a.1, b.1 and c.1 correspond to the cross-section near the membrane surface, and images a.2, b.2 and c.2 correspond to the interior of the membranes.

2. Refractometry test for the estimation of the saturation NP concentration (C_{sat})

Refractometry technique was used to estimate the nanoparticle (NP) concentration from which the solution of chitosan (CS) with NP was saturated. This technique allows the measurement of the refractive limit angle (or critical angle) (Θ) created at the interface between a solution and the refractometer prism (i.e., the smallest angle of incidence that produces a total internal reflection). Different concentrations of each NP (0.5%, 2%, 5%, 9%, 17%, 33%, 41% and 50% w/w) in the CS solution were considered. When the CS solution with NPs begins to be saturated, the critical angle tends to a stable critical angle value, indicating that the solution does not admit more NPs.

The measurements were made with an Abbe refractometer model Bellingam & Stanley Ld. Critical angle 60/ED (Tunbridge Wells, United Kingdom), with an accuracy of 0.1 arc minutes, and with a refractive index of the refractometer prism n_p = 1.76141. The direct reading gave the critical

angle (Θ_C) of the solution with respect to the prism of the refractometer. The monochromatic light source used to illuminate the refractometer was a sodium lamp (Na), with a 589 nm wavelength.

The obtained critical angles of the CS solutions containing the metallic oxide NPs (TiO_2, Fe_3O_4, Al_2O_3) were plotted in **Figure S4** as a function of the NP concentration. The change of the tendency of the critical angle with the increase of the NP concentration indicated an excess of the NP in the CS solution. In other words, from the intersection of the two straight lines the maximum NP concentration in the CS solution corresponding to the saturation concentration can be determined.

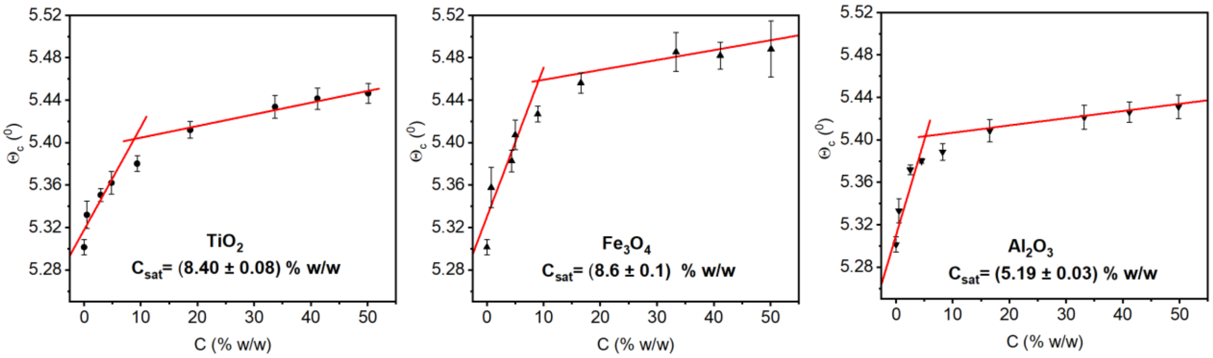

Figure S4: Critical angle (Θ_C) as a function of the NP concentration in the CS solution: TiO_2 (●), Fe_3O_4 (▲) and Al_2O_3 (▼).

Good and stable CS-NP dispersions could be prepared when the TiO_2 and Fe_3O_4 concentrations in the CS solutions were around 8% w/w, and when the Al_2O_3 concentration was around 5% w/w. These agree with the obtained saturation concentrations (C_{sat}) from the data plotted in **Figure S4**. It must be mentioned that 24 h after the addition of the NP in the CS solution, agglomeration followed by sedimentation of TiO_2 and Fe_3O_4 started to occur when the concentrations of these NPs were higher than 41% w/w. The same occurred for the Al_2O_3 in the CS solutions when the NPs concentration was greater than 33% w/w. Therefore, in the present research study, CS-based composite membranes were prepared with NPs concentrations below and the above cited C_{sat} values. Low NP concentration was considered as the prepared NP concentration (0.5%, 2%, 5%, 9%, 17%, 33%, 41% or 50% w/w) immediately lower than the Csat value to sure that no NP saturation takes place. On the hand, high NP concentration was considered as the prepared NP concentration (0.5%, 2%, 5%, 9%, 17%, 33%, 41% or 50% w/w) higher than the Csat value but lower than NP concentration where sedimentation was observed. Thus, we could be sure that NP saturation take place but not sedimentation. The NPs concentrations 5 % w/w (low concentration) and 33% w/w (high concentration) were selected for TiO_2 and Fe_3O_4, whereas 2% w/w (low concentration) and 17% w/w (high concentration) were selected for Al_2O_3.

3. Effects of TPP post-treatment on the mechanical properties of CS-NP composite membranes

The effect of the applied TPP post-treatment on the CS-NP composite membranes modified their mechanical properties, as shown in **Table S2**. As it was expected, an improvement of the mechanical properties was observed when the NP concentration increased being greater for the CS-NP composite membrane with TiO_2. A significant decrease of the elongation at break (ε_b) of the CS membrane was observed after adding the NPs and after applying the TPP post-treatment. The TPP post-treatment of the CS-NP composite membranes slightly reduced the elongation at break. The increase of E of the CS-TiO_2 membrane after the post-treatment was significantly higher (48%) than that of the CS membrane prepared without NPs, whereas the increase of E was lower for the CS-Fe_3O_4 membrane (35%) and for the CS-Al_2O_3 membrane (29%). On the other hand, there is a considerable increase of

the tensile strength (τ_s) of the CS-NP composite membranes after the TPP post-treatment with respect to the CS membrane prepared without NPs. This was more noticeable at high NP concentration, which is 53% for the CS-TiO_2 membrane, 17% for the CS-Fe_3O_4 membrane, and 26% for the CS-Al_2O_3 membranes. This behaviour was expected, since the TPP reticulates the chains of CS and immobilizes the dispersed NPs in its network, obtaining membranes with a greater tenacity by increasing their tensile strength and reducing their elasticity.

Table S2: Mechanical properties (Young's modulus, *E*; tensile strength, τ_s; elongation at break, ε_b), of the CS membranes prepared with and without NPs, and with and without 3% w/w TPP post-treatment for 20 h at pH 4.

Membrane	*Without TPP post-treatment*			*With TPP post-treatment*		
	E *(GPa)*	τ_s *(MPa)*	ε_b *(%)*	*E* *(GPa)*	τ_s *(MPa)*	ε_b *(%)*
CS	2.5 ± 0.2	86 ± 10	48 ± 7	4.8 ± 0.6	114 ± 6	7.9 ± 0.4
CS-TiO_2-L	3.0 ± 0.3	70 ± 9	8 ± 2	6.7 ± 0.4	122 ± 6	3.5 ± 0.3
CS-TiO_2-H	4.0 ± 0.3	72 ± 8	7 ± 2	7.1 ± 0.5	174 ± 24	2.6 ± 0.4
CS-Fe_3O_4-L	3.2 ±0.3	70 ± 3	6.7 ± 0.5	5.1 ± 0.6	117 ± 16	7.2 ± 0.9
CS-Fe_3O_4-H	4.0 ± 0.4	77 ± 10	4.5 ± 0.6	6.5± 0.3	133 ± 8	4.1 ± 0.5
CS-Al_2O_3-L	3.3 ± 0.3	71 ± 8	11 ± 3	5.4 ± 0.5	121 ± 10	4.0 ± 0.9
CS-Al_2O_3-H	4.0 ± 0.3	77 ± 7	6 ± 1	6.2 ± 0.2	144 ± 17	3.0 ± 0.7

4. Study of the interaction between ASA and CS-NP composite membranes

The final pH values of both membrane sides after carrying out the ASA transport experiments (feed and permeate containers) are listed in **Table S3**.

Table S3: Final pH values of the feed solution after carrying out the ASA transport experiments with the simulated gastric fluid (SGF), simulated intestinal fluid (SIF) and simulated gastrointestinal transit medium (SGIT): pH_f is the final pH value of the feed solution that initially contained ASA, and pH_P is the final pH value of the permeate solution.

**Membrane**	**SGF**		**SIF**		**SGIT**	
	**pH_f**	**pH_P**	**pH_f**	**pH_P**	**pH_f**	**pH_P**
CS	1.70	1.33	3.24	6.70	2.53	6.11
CS-TiO_2-L	1.64	1.44	3.54	6.60	3.68	6.32
CS-TiO_2-H	1.65	1.45	3.45	6.67	2.88	6.50
CS-Fe_3O_4-L	1.62	1.67	3.39	6.60	3.47	6.25
CS-Fe_3O_4-H	1.51	1.51	3.08	6.69	3.10	6.25
CS-Al_2O_3-L	1.55	1.40	4.54	6.39	3.41	6.7
CS-Al_2O_3-H	1.43	1.36	4.50	6.41	4.22	6.18

In all the experiments, the initial pH value of the feed solution was pH_{ASA} = 2.6. For the permeate side, the initial pH value varied depending on the simulated fluids: the initial pH value was 1.2 in both the simulated gastric fluid (SGF) and the simulated gastrointestinal transit medium (SGIT), whereas in the simulated intestinal fluid (SIF) the pH was 6.8. As can be seen in **Table S3** the feed pH was changed at the end of the experiments. For the SGF experiments, the final pH of the feed solution was decreased, whereas these values increased for SIF and SGIT experiments. This change of the final

pH of the feed solution indicates that a reverse flow (from the permeate to feed side) occurred. This is due to the high swelling degree of the used membranes. A greater reverse flow resulted in a higher pH change decreasing the ASA release velocity.

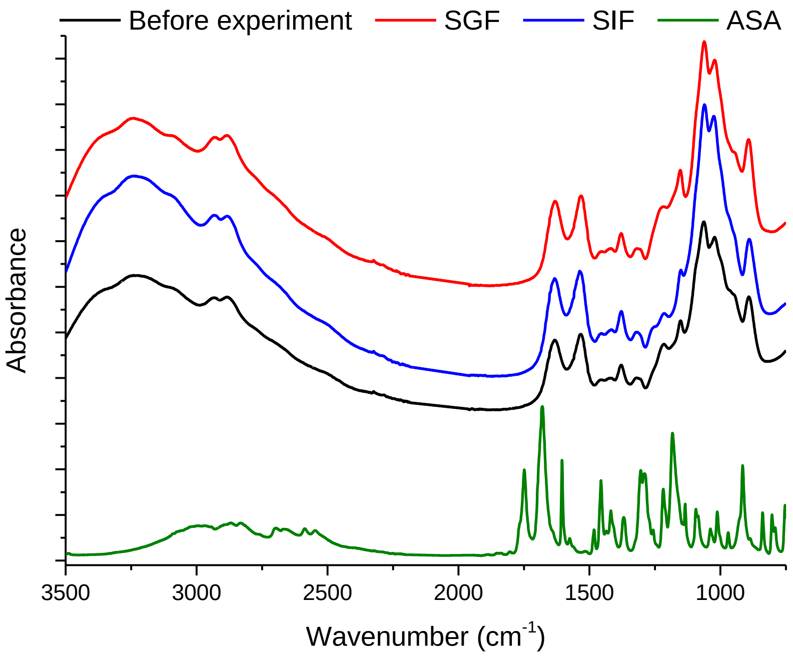

Figure S5: FTIR spectra of the CS membranes prepared without NP before (black line) and after the transport experiments using different permeate media: SGF with pH_{SGF} = 1.2 (red line) and SIF with pH_{SIF} = 6.8 (blue line). For sake of comparison, the FTIR spectrum of ASA (green line) is also shown.

Figure S5 shows the FTIR spectra of the CS membrane before and after the ASA transport as well as the FTIR spectrum of the ASA. No interaction between ASA and the CS membrane was confirmed by the absence of ASA peaks in the FTIR spectra of the CS membrane after the ASA transport experiments.

References

1. Mi, F.L.; Shyu, S.S.; Lee, S.T.; Wong, T.B. Kinetic study of chitosan-tripolyphosphate complex reaction and acid-resistive properties of the chitosan-tripolyphosphate gel beads prepared by in-liquid curing method. *J. Polym. Sci., Part B: Polym. Phys.* **1999**, *37*, 1551-1564.
2. Paños, I.; Acosta, N.; Heras, A. New drug delivery system based on chitosan. *Curr. Drug Discov. Technol.* **2008**, *5*, 333-341.
3. Velickova, E.; Winkelhausen, E.; Kuzmanova, S.; Alves, V.D.; Moldão-Martins, M. Impact of chitosan-beeswax edible coatings on the quality of fresh strawberries (fragaria ananassa cv camarosa) under commercial storage conditions. *Food Sci. Technol. Int.* **2013**, *52*, 80-92.
